# Regulatory T cells are paramount effectors in progesterone regulation of embryo implantation and fetal growth

**DOI:** 10.1172/jci.insight.162995

**Published:** 2023-06-08

**Authors:** Ella S. Green, Lachlan M. Moldenhauer, Holly M. Groome, David J. Sharkey, Peck Y. Chin, Alison S. Care, Rebecca L. Robker, Shaun R. McColl, Sarah A. Robertson

**Affiliations:** 1 Robinson Research Institute, School of Biomedicine, and; 2 School of Biological Sciences, University of Adelaide, Adelaide, South Australia, Australia.

**Keywords:** Reproductive Biology, Embryonic development, T cells, Tolerance

## Abstract

Progesterone (P4) is essential for embryo implantation, but the extent to which the pro-gestational effects of P4 depend on the maternal immune compartment is unknown. Here, we investigate whether regulatory T cells (Treg cells) act to mediate luteal phase P4 effects on uterine receptivity in mice. P4 antagonist RU486 administered to mice on days 1.5 and 3.5 postcoitum to model luteal phase P4 deficiency caused fewer CD4^+^Foxp3^+^ Treg cells and impaired Treg functional competence, along with dysfunctional uterine vascular remodeling and perturbed placental development in midgestation. These effects were linked with fetal loss and fetal growth restriction, accompanied by a Th1/CD8-skewed T cell profile. Adoptive transfer at implantation of Treg cells — but not conventional T cells — alleviated fetal loss and fetal growth restriction by mitigating adverse effects of reduced P4 signaling on uterine blood vessel remodeling and placental structure and by restoring maternal T cell imbalance. These findings demonstrate an essential role for Treg cells in mediating P4 effects at implantation and indicate that Treg cells are a sensitive and critical effector mechanism through which P4 drives uterine receptivity to support robust placental development and fetal growth.

## Graphical abstract

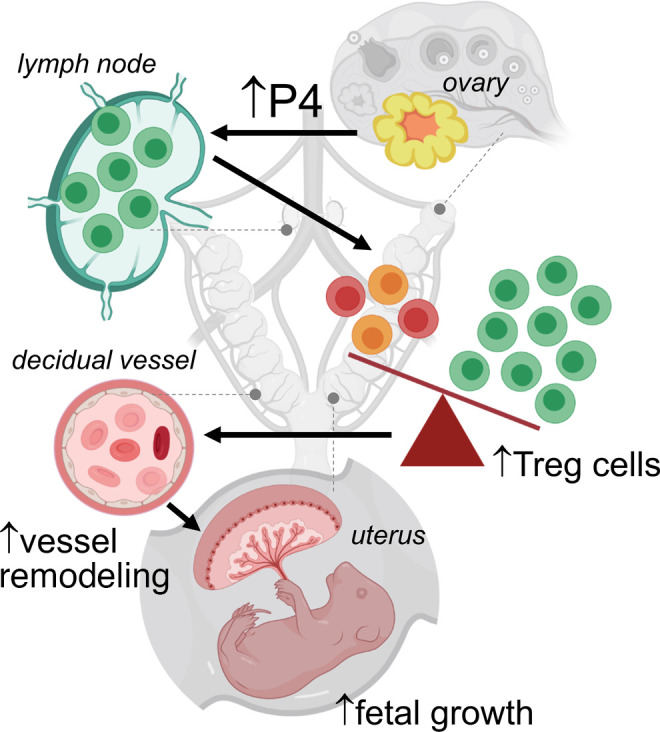

## Introduction

The sex steroid hormone progesterone (P4) is secreted by the corpus luteum of the ovary in the luteal phase of the menstrual cycle and is essential for initiating and maintaining pregnancy (1–3). Sufficient luteal P4 signaling is critical for embryo implantation and is implicated as a determinant of robust placental development and healthy fetal growth (4, 5). P4 actions include transcriptional reprogramming of uterine epithelial and stromal cells to induce endometrial receptivity to embryo attachment and to promote decidual transformation of stromal fibroblasts (6), events that are essential to allow implantation and trophoblast invasion and underpin healthy placental development (4, 7). These cellular changes are accompanied by concerted adaptations in the maternal immune response to induce a state of immune tolerance that suppresses inflammation and protects the semi-allogeneic embryo from detrimental effects of immune effector cells (8–10). Reduced luteal P4 bioavailability is implicated in the pathophysiology of unexplained infertility and recurrent miscarriage, due to inadequate corpus luteum P4 secretion or low uterine responsiveness to P4 giving rise to “luteal phase deficiency” (3, 11, 12). Luteal phase deficiency is thought to be a factor underpinning “shallow placentation,” which predisposes to fetal loss and disorders that manifest in later gestation, particularly fetal growth restriction and preeclampsia (3, 7, 13–16).

P4 signaling effects on receptivity to implantation are mediated largely through effects on uterine stromal cells (17). They express canonical P4 receptor (PR) and undergo extensive transcriptional changes upon P4-induced decidual transformation that are essential to placental trophoblast invasion (1, 6). Immune cells may also express PRs and acquire different functional states in response to P4 signaling (18–20). The extent to which immune cells in the uterus mediate P4 effects at implantation is unknown and important to define, as many disorders of pregnancy exhibit inflammatory features and have an underlying immune etiology (21, 22). These conditions are common and debilitating (23, 24), but their pathophysiology is poorly understood. There is an imperative to understand the underlying causes and develop preventative interventions.

CD4^+^Foxp3^+^ regulatory T (Treg) cells are central mediators of pregnancy tolerance through their potent immune-regulatory and antiinflammatory activity and are a top candidate for P4-mediated actions via the immune response. In women, reduced uterine Treg cell abundance and/or altered phenotype are implicated in the pathophysiology of infertility (25), as well as recurrent miscarriage (26, 27), fetal growth restriction, preeclampsia, and spontaneous preterm birth (28–31). Reproductive disorders arising from impaired Treg cells are associated with uncontrolled pro-inflammatory CD4^+^ or CD8^+^ T effector (Teff) cell responses to fetal alloantigens (32–35). Mouse studies are an informative model of immune disorders of pregnancy and confirm that semi-allogeneic fetuses cannot survive without sufficient maternal Treg cells (10, 36–38) to suppress Teff cells that otherwise cause pregnancy failure (39, 40). In addition, decidual Treg cells interact with fetal trophoblast cells and engage with uterine NK (uNK) cells, macrophages, dendritic cells, and mast cells to modulate their phenotypes toward regulatory and tolerogenic functions (41–46). Through effects on the uterine immune network, Treg cells influence the remodeling of the uterine vasculature required for optimal placental development (47), without which placental blood flow and fetal growth are compromised (48, 49).

Recent studies strongly implicate P4 in Treg cell proliferation, phenotype commitment, and suppressive function (18, 50–53). The peri-conception phase is critical for the generation of sufficient Treg cells to persist over the course of pregnancy. Treg cells in the uterine decidua in women show evidence of antigen-specific induction and proliferation at the outset of pregnancy (54, 55). This can be modeled in mice, where Treg cells are recruited into the uterine decidua from the peripheral blood during early pregnancy after expansion of the Treg pool in uterine draining lymph nodes, in response to sex steroid hormones and antigens delivered in seminal fluid at conception (10, 56).

These observations raise the question of the degree to which Treg cells mediate the impact of luteal phase P4 and contribute to pathophysiological mechanisms, linking altered luteal phase P4 signaling with adverse pregnancy outcomes. Here, we employed a mouse model that mimics human luteal phase insufficiency using low-dose P4 antagonist RU486. Our results demonstrate that Treg cells are highly sensitive to luteal phase P4 and a key mechanism by which luteal P4 bioavailability affects implantation success, placental development, and fetal growth and survival in late gestation.

## Results

### Impaired luteal phase P4 signaling causes fetal growth restriction and fetal loss.

**Figure 1:**
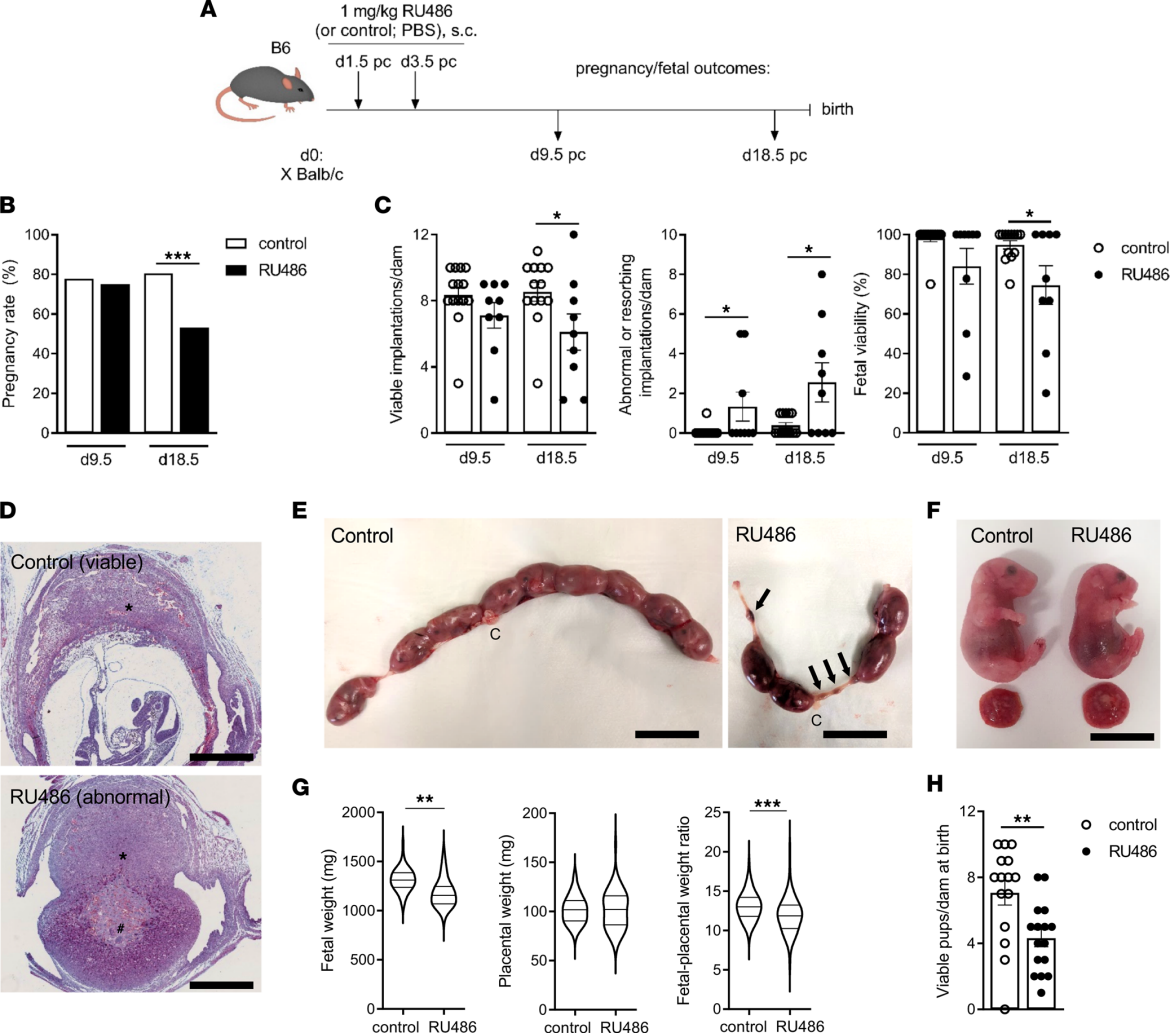
Impaired luteal phase P4 signaling causes fetal loss and fetal growth restriction in late gestation. Female C57BL/6 (B6) mice were mated to BALB/c males and administered RU486 (1 mg/kg) or vehicle (control) on 1.5 and 3.5 dpc. Pregnancy and fetal outcomes were assessed in treated mice at 9.5 and 18.5 dpc and at birth. (**A**) Schematic of experimental design. (**B**) Pregnancy rate (% mated mice with ≥1 implantation site at 9.5 dpc or fetus at 18.5 dpc). (**C**) Number of normal implantation sites or viable fetuses per pregnant dam, number of abnormal or resorbing implantation sites (fetal losses) per pregnant dam, and fetal viability as percentage total implantation sites per pregnant dam were measured. See Supplemental Figure 1 for images of uteri recovered at 9.5 dpc. (**D**) Representative photomicrographs of viable implantation sites from control pregnant dams and abnormal implantation sites from RU486-treated dams on 9.5 dpc, stained with Masson’s trichrome. Asterisk indicates decidua. Pound sign indicates degenerating fetal tissue. Scale bar = 1 mm. See Supplemental Figure 2 for additional histology of implantation sites. (**E**) RU486-treated dams exhibit an elevated fetal resorption rate on 18.5 dpc. Resorption sites are indicated by arrows; letter C indicates cervix. Scale bar = 20 mm. (**F**) Fetuses of RU486-treated dams are visibly growth restricted. Scale bar = 12 mm. (**G**) Fetal and placental weights and fetal weight/placental weight ratios were measured in viable fetuses on 18.5 dpc. (**H**) In a separate cohort, mice were allowed to deliver, and number of viable pups per dam was quantified. For panels **B**, **C**, **G**, and **H**, treatment group is indicated in legend. (**B**) *n* = 36–49 mated females/group; data analyzed by χ^2^ test. (**C** and **H**) *n* = 9–16 pregnant dams/group; data shown as mean ± SEM with individual mice indicated by symbols, analyzed by unpaired *t* test; (**G**) *n* = 70–110 fetuses or placentas/group; data shown as violin plots with median and quartile values marked, analyzed by linear mixed model ANOVA with mother as subject. *P < 0.05, **P < 0.01, ***P < 0.001.

Initially we established a model of impaired luteal phase P4 signaling to investigate the consequences of reduced P4 signaling on embryo implantation and late gestation pregnancy outcomes and to define the role of an altered Treg cell pool in any effects. To achieve this, C57BL/6 female mice were mated to BALB/c males and administered P4 antagonist RU486 (mifepristone) at a range of doses (0.5–8 mg/kg) on both 1.5 and 3.5 days post coitum (dpc) (Figure 1A), a time comparable to early and mid-luteal phase (~2–6 days after the peri-ovulatory surge in luteinizing hormone, LH) in women (57, 58). When embryo implantation was assessed in midgestation at 9.5 dpc, a dose-dependent effect of RU486 treatment on implantation rate was observed (Supplemental Figure 1; supplemental material available online with this article; https://doi.org/10.1172/jci.insight.162995DS1). As expected (52, 58, 59), complete implantation failure was evident in mice administered 8 mg/kg RU486, but implantation was progressively less impaired with lower RU486 doses. Most dams given 1 mg/kg RU486 were pregnant and showed normal implantation rates (Figure 1, B and C). While the majority of implantation sites appeared viable after administration of 1 mg/kg RU486 (Figure 1C), a higher proportion were abnormal (small and/or avascular) compared with those in control dams (Supplemental Figure 1). Histological assessment showed these abnormal sites usually contained decidual tissue, but placentation was impaired and fetal demise was evident (Figure 1D and Supplemental Figure 2).

At 18.5 dpc, mice given 1 mg/kg RU486 showed evidence of adverse pregnancy outcomes. Pregnancy rate was reduced by 28% (RU486 group; 26/49 mice had ≥1 viable fetus, versus control group; 29/36; *P* < 0.001) (Figure 1B). Mice that were pregnant (dams) had on average 20% fewer viable fetuses and increased fetal loss (resorption) (Figure 1, C and E). Among viable fetuses of dams given RU486, fetal growth restriction was evident, with mean fetal weight reduced by 15% (*P* < 0.01), along with a decrease in the fetal/placental weight ratio, indicating reduced placental transport efficiency (*P* < 0.001) (Figure 1, F and G). In a second cohort, when RU486-treated dams progressed to birth, there was a 40% decline in the number of viable pups born compared with controls (*P* < 0.01) (Figure 1H). Together, these data demonstrate that impaired luteal phase P4 signaling caused by low-dose (1 mg/kg) RU486 has only a modest effect on implantation but a substantial adverse impact on progression of pregnancy reflected in late gestation parameters — giving rise to pregnancy loss, fetal resorption, fetal growth restriction, and reduced perinatal fetal viability. Therefore, this RU486 treatment regimen was used for subsequent experiments to investigate the significance of Treg cells in adverse outcomes caused by impaired luteal phase P4 signaling.

### Impaired luteal phase P4 signaling disrupts Treg cell generation in uterus-draining lymph nodes.

**Figure 2:**
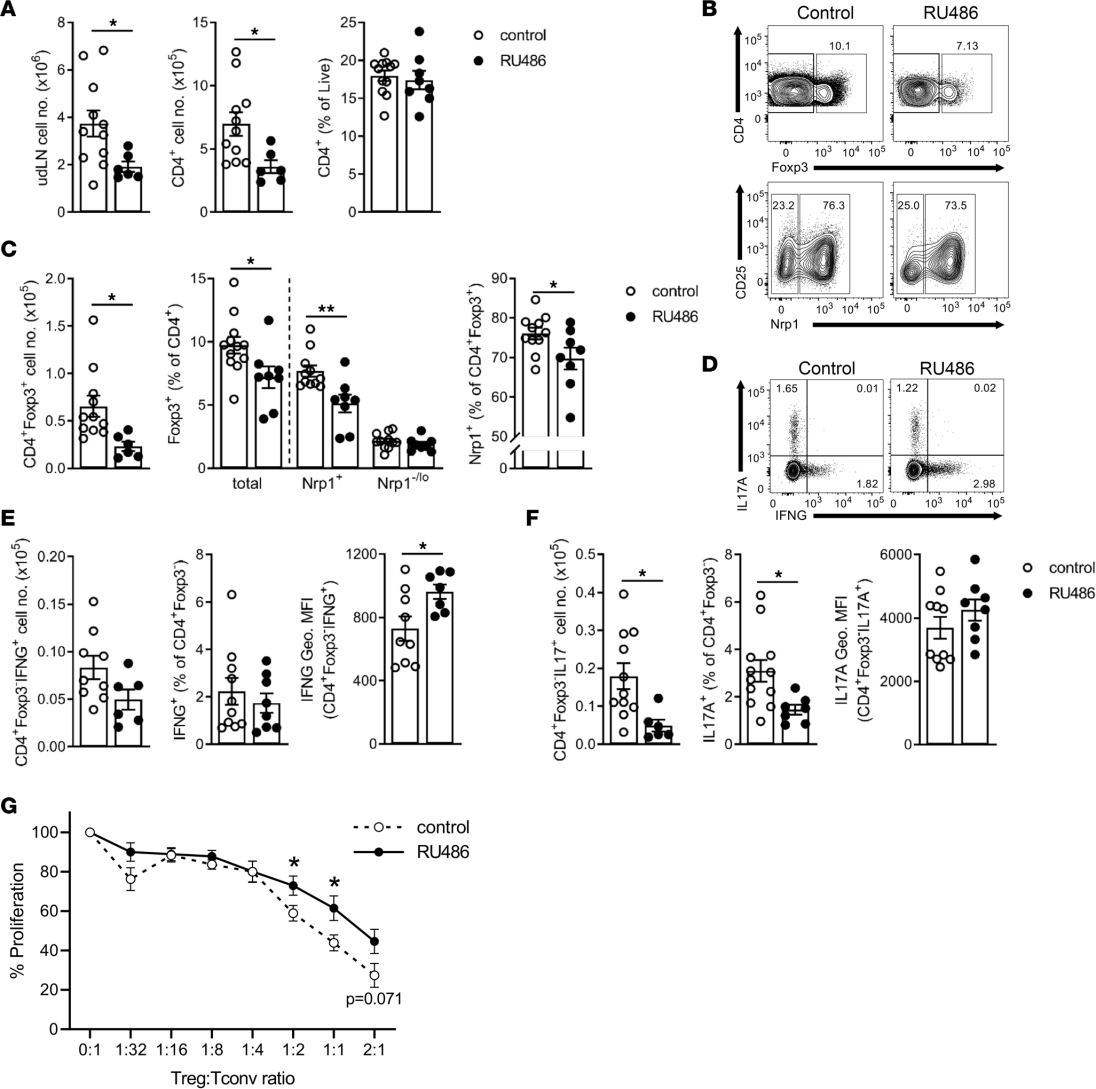
Impaired luteal phase P4 signaling causes CD4^+^ Treg cell deficiency in udLNs in midgestation. Female C57BL/6 (B6) mice were mated to BALB/c males and administered RU486 (1 mg/kg) or vehicle (control) on 1.5 and 3.5 dpc, and then udLNs were excised from pregnant (≥1 viable implantation site) mice on 9.5 dpc. (**A**) Total cell count and number and proportion of CD4^+^ T cells in the udLNs of control and RU486-treated mice. (**B**) Representative FACS plots of Foxp3 staining in CD4^+^ T cells and Nrp1 staining in CD4^+^Foxp3^+^ T cells in the udLNs of control and RU486-treated mice. Among CD4^+^ T cells, Treg cells were defined as Foxp3^+^, thymic derived Treg (tTreg) cells were classified as Foxp3^+^Nrp1^+^, and peripherally induced Treg cells were classified as Foxp3^+^Nrp1^–/lo^. (**C**) Quantification of Foxp3^+^ Treg cell number; proportions of Foxp3^+^, Foxp3^+^Nrp1^+^, and Foxp3^+^Nrp1^–/lo^ Treg cells (%CD4^+^ cells); and proportion of Foxp3^+^Nrp1^+^ cells (%Foxp3^+^ cells). (**D**) Representative FACS plots of IFNG and IL-17A staining in udLN CD4^+^Foxp3^–^ T cells from control and treated mice. (**E** and **F**) Number and proportion (of CD4^+^ cells) of IFNG^+^ (Th1) cells (**E**) and IL-17^+^ (Th17) cells (**F**). Also shown is the geometric MFI of IFNG in Th1 cells (**E**) and IL-17 in Th17 cells (**F**). (**G**) Ex vivo analysis of suppressive activity in Treg (CD4^+^CD25^+^) cells isolated and pooled from udLNs of 1–3 pregnant control or RU486-treated mice on 8.5–9.5 dpc and coincubated with responder spleen conventional T (Tconv; CD4^+^CD25^–^) cells. Tconv cell proliferation was determined by CFSE staining and flow cytometry analysis. Proliferation of Tconv cells (%control, no Treg cells) at each Treg/Tconv ratio is depicted. (**A**, **C**, **E**, and **F**) *n* = 6–15 pregnant dams/group; data shown as mean ± SEM with individual mice indicated by symbols. (**G**) *n* = 9–10 cell pools/group in 7 experimental replicates; data shown as mean ± SEM with mean of biological replicates indicated by symbols. (**A**, **C**, **E**, **F**, and **G**) Data were analyzed by unpaired *t* test; *P < 0.05, **P < 0.01.

The uterus-draining para-aortic lymph nodes (udLNs) are the main site of T cell generation in pregnancy, and many studies confirm a typical 2- to 4-fold expansion in the Treg cell pool by 9.5 dpc compared with nonpregnant controls (37, 60, 61). To evaluate the effect of reduced P4 bioavailability on the Treg cell pool, we analyzed Treg cells (defined as CD4^+^Foxp3^+^ cells) in the udLNs of RU486-treated dams on 9.5 dpc by flow cytometry. The udLNs of RU486-treated dams contained approximately 50% fewer total cells, due primarily to an approximately 50% reduction in total CD4^+^ T cells (Figure 2A) and 20% reduction in CD8^+^ T cells (Supplemental Figure 3). Strikingly, Foxp3^+^ Treg cells were differentially affected, so the mean proportion of Treg cells among CD4^+^ T cells was reduced from 10% to 7% in dams given RU486. This was attributable primarily to a 30% smaller proportion of neuropilin 1–positive (Nrp1^+^) thymus-derived Treg (tTreg) cells (*P* < 0.01) (Figure 2, B and C). The mean total number of Treg cells in udLNs of RU486-treated mice was decreased by 64% in RU486-treated mice compared with controls (*P* < 0.05).

Teff cells in udLNs were also assessed following RU486 treatment. The number and proportion of IL-17–producing CD4^+^ T cells (Th17 cells) were decreased relative to total CD4^+^ T cells (*P* < 0.05) (Figure 2, D and F). Although the number of CD4^+^IFNG^+^ T cells (Th1 cells) was unchanged (Figure 2E), the MFI of IFNG in Th1 cells was increased, reflecting enhanced IFNG expression per cell following RU486 treatment (*P* < 0.05) (Figure 2E). The proportion of IFNG-expressing cytotoxic CD8^+^ T (Tc1) cells was unchanged in the udLNs (Supplemental Figure 3B). However, the CD8/Treg ratio was increased following RU486 treatment (Supplemental Figure 3C), highlighting an overall shift toward pro-inflammatory CD8^+^ T cell immunity due to impaired P4 signaling. This shift was likely secondary to the loss of Treg cells and exacerbated by altered Treg cell suppressive competence, as demonstrated by reduced capacity of Treg cells from dams administered RU486 to suppress responder Tconv cell proliferation in ex vivo suppression assays (Figure 2G).

The effect of RU486 on Treg cells in midgestation was not limited to the udLN, as RU486-treated mice also had fewer Treg cells in the spleen (*P* < 0.05) (Supplemental Figure 5B). In addition, there were decreased CD4^+^ T cells (Supplemental Figure 5A), a trend toward increased CD8^+^ T cells (Supplemental Figure 3D), and an increased proportion of IFNG-expressing Tc1 cells (*P* < 0.01) (Supplemental Figure 3E). However the degree of Treg cell loss was not as extensive (mean 44% loss in spleen vs. 64% loss in udLNs), and suppressive competence of splenic Treg cells appeared unaffected by RU486 treatment (Supplemental Figure 5C).

Several previous studies show that Treg cells proliferate in the udLN over the course of early pregnancy to increase 2- to 3-fold by implantation and to peak in midgestation (60, 62). When T cell populations in the udLN were examined at the time implantation commences on 4.5 dpc, just 24 hours following the second dose of RU486, CD4^+^ T cells were already fewer in number compared with control mice (Supplemental Figure 4, C–E), implying that the smaller Treg cell pool in midgestation arises due to impaired proliferation of udLN Treg cells from early pregnancy.

Together these data demonstrate that peri-implantation P4 signaling is essential to support development of a robust Treg cell population during early pregnancy and to constrain proliferation of pro-inflammatory Th1 and Tc1 cells. As Treg cells limit antifetal inflammation and are required for normal fetal growth, this raises the question of whether reduced Treg cells and increased Th1 immunity are causal, or simply bystander effects, in the adverse pregnancy outcomes observed in mice with luteal phase P4 signaling disruption.

### P4 directly regulates the phenotype of Treg and Teff cells through suppression of IFNG.

To further investigate how P4 alters the Treg cell response, we examined whether P4 has a direct effect on Treg cell phenotype in vitro. Given evidence that Treg cells express PR (18) and our finding of increased expression of IFNG by T cells after RU486 administration in vivo, we postulated that P4 controls T cell phenotype stability, to strengthen Treg cell functional capacity and decrease effector cell immunity.

**Figure 3:**
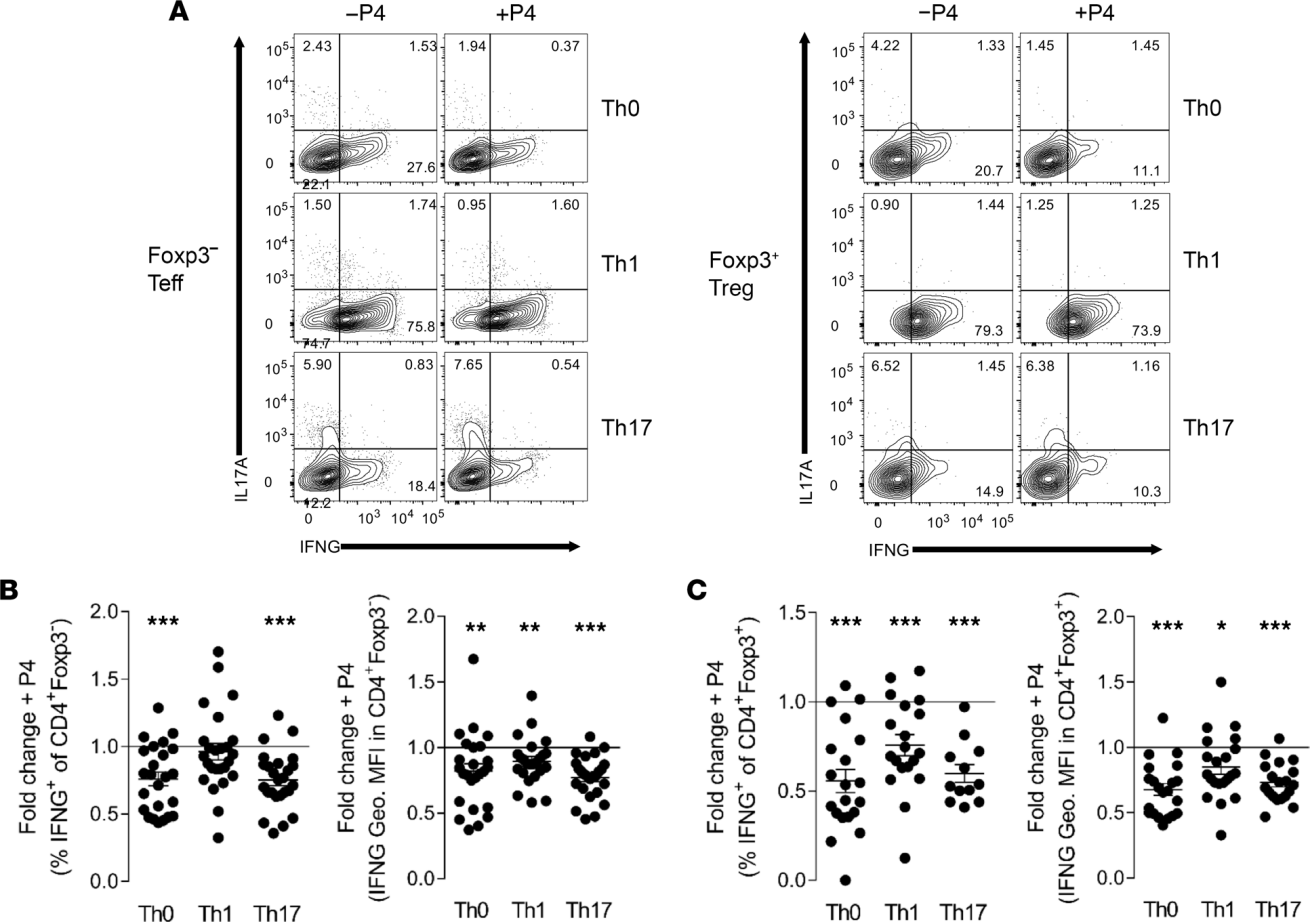
P4 suppresses IFNG production in Teff and Treg cells in vitro. Splenocytes from B6 female mice in estrus were cultured under Th1-polarizing, Th17-polarizing, or nonpolarizing conditions in the presence or absence of P4 (0.5 μg/mL) for 48 hours followed by stimulation with PMA and ionomycin for 4 hours and subsequent quantification of Teff and Treg cell cytokine production by flow cytometry. (**A**) Representative flow cytometric analysis of IFNG expression in (**B**) Teff (CD4^+^Foxp3^–^) and (**C**) Treg (CD4^+^Foxp3^+^CD25^+^) cells and IL-17A expression in (**B**) Teff and (**C**) Treg cells, cultured under Th0-, Th1-, or Th17-polarizing conditions in the presence or absence of P4. (**B** and **C**) Proportion and geometric MFI of IFNG in (**B**) Teff cells and (**C**) Treg cells, expressed as fold-change in +P4 compared with respective –P4 control. (**B** and **C**) *n* = 15–21 mice/group, in 5 individual experiments. Each symbol represents an individual mouse. Data are shown as mean fold-change ± SEM. Data were analyzed by 1-tailed *t* test where –P4 control = 1.0; *P < 0.05, **P < 0.01, ***P < 0.001.

To determine whether P4 has a direct effect on Treg cell stability/phenotypic plasticity in vitro, we measured CD4^+^ Treg and Teff cell cytokine-secreting potential under pro-inflammatory Th1- and Th17-polarizing conditions in the absence or presence of P4, at a concentration approximating physiological levels in pregnancy (0.5 μg/mL). Splenocytes from B6 female mice in estrus were activated using plate-bound anti-CD3 (α-CD3) and soluble α-CD28 under nonpolarizing (Th0-), Th1-, or Th17-polarizing conditions, with the addition of P4 (0.5 μg/mL) or control. After 48 hours, cells were removed from cultures, and Treg and Teff cell proportion and phenotype, including IFNG and IL-17A cytokine production, were analyzed by flow cytometry. The addition of P4 to cultures caused suppression of IFNG production in both Treg and Teff cells cultured under standard Th0 conditions and under conditions polarizing toward either Th1 or Th17 cell differentiation (Figure 3, A–C). Conversely, IL-17 production in Teff and Treg cells was unchanged in all conditions tested (Supplemental Figure 6, A and B).

These results build on previous findings that P4 suppresses Th1 cells in vitro (63, 64), to show that P4 also exerts strong polarizing effects in Foxp3^+^ Treg cells. These data support the interpretation that in vivo, P4 directly influences the phenotypic plasticity of Treg cells to reinforce Treg cell fate commitment, explaining why reduced P4 signaling during early pregnancy impairs Treg cell generation and suppressive function and causes Th1 cells to exhibit enhanced IFNG expression in midgestation.

### Adoptive transfer of Treg cells to mice following impaired luteal phase P4 signaling improves pregnancy outcomes and restores normal fetal growth.

Since Treg cells were diminished in midgestation following impaired luteal phase P4 signaling, and insufficiency in Treg cells is known to cause pregnancy loss and fetal growth restriction in later gestation (36, 37, 39, 65), we hypothesized that loss of Treg cells was causally involved in the poor pregnancy outcome observed after RU486 administration. To test this, we adoptively transferred Treg cells to mated mice treated as before with 1 mg/kg RU486, then measured maternal and fetal parameters in late gestation. CD4^+^CD25^+^ (Treg) and CD4^+^CD25^–^ (Tconv) cells were isolated from spleen and lymph nodes (LNs) of BALB/c-mated donor B6 females on 11.5–14.5 dpc and were administered i.v. to RU486-treated recipient B6 mice approximately 8 hours following RU486 administration on 3.5 dpc (Supplemental Figure 7A). In some cases, Pepcb/BoyJ (CD45.1) congenic females were used as donors to enable the detection of transferred cells in B6 recipients (CD45.2), which were evident in the udLN 72 hours after transfer (Supplemental Figure 7B).

**Figure 4:**
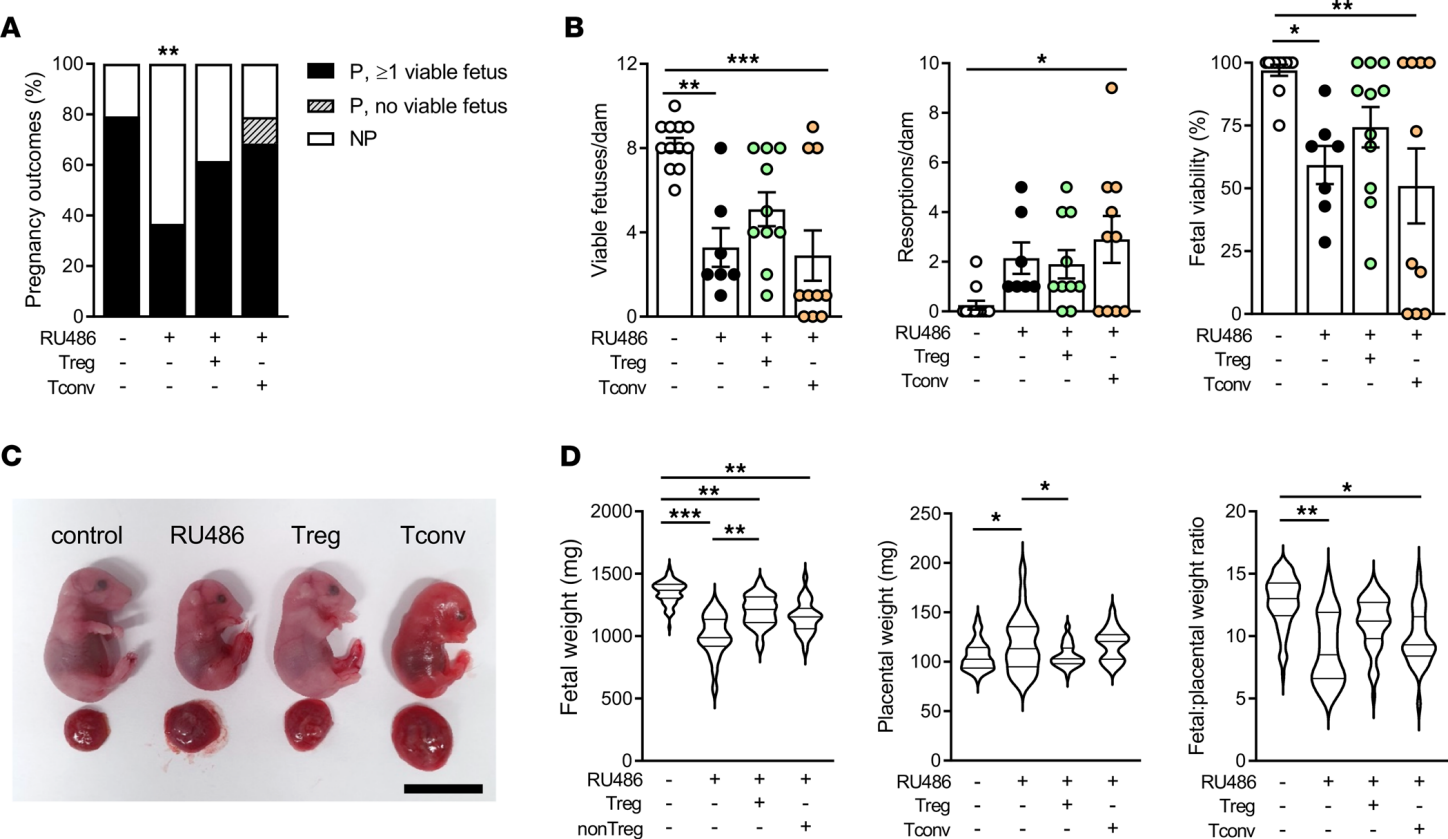
Treg cell transfer restores fetal loss and fetal growth restriction caused by impaired luteal phase P4 signaling. Female B6 mice were mated to BALB/c males and administered RU486 (1 mg/kg) or vehicle (control) on 1.5 and 3.5 dpc. On 3.5 dpc, approximately 8 hours following the final RU486 dose, females were injected i.v. with 2 × 10^5^ Treg cells (CD4^+^CD25^+^), Tconv cells (CD4^+^CD25^–^), or vehicle control (PBS). On 18.5 dpc maternal and fetal outcomes were measured. (**A**) Pregnancy outcomes for dams treated with control, RU486, RU486+Treg cells, or RU486+Tconv cells, classified as pregnant with ≥1 viable fetus, pregnant with only nonviable fetuses, or nonpregnant. P, pregnant; NP, nonpregnant. (**B**) Number of viable fetuses, number of resorptions, and percentage fetal viability in pregnant dams (with ≥1 viable fetus) treated with control, RU486, RU486+Treg cells, and RU486+Tconv cells. (**C**) Representative images of fetuses and placentas from control dams and dams given RU486, RU486+Treg cells, or RU486+Tconv cells. Scale bar = 12 mm. (**D**) Fetal weight, placental weight, and fetal/placental weight ratio were measured in viable fetuses. (**A**) *n* = 19–30 mated females/group; data analyzed by χ^2^ test comparing pregnant and nonpregnant mice. (**B**) *n* = 7–12 pregnant dams/group; mean ± SEM with individual dams indicated by symbols; data analyzed by 1-way ANOVA with Sidak’s post hoc *t* test. (**D**) *n* = 27–89 fetuses or placentas/group; data shown as violin plots with median and quartile values marked, analyzed by linear mixed model ANOVA with mother as subject. *P < 0.05, **P < 0.01, ***P < 0.001.

RU486-treated dams evaluated on day 18.5 dpc again showed a reduced pregnancy rate compared with controls, had fewer viable fetuses per pregnant dam (Figure 4, A and B), and exhibited fetal growth restriction (Figure 4, C and D). In contrast, the mice administered Treg cells had a similar pregnancy rate (Figure 4A) and comparable fetal viability to controls (Figure 4B). Notably, fetal weight in dams administered Treg cells was significantly improved compared with RU486-treated mice given PBS, and the placental weight and fetal/placental weight ratio was not different from control pregnancies (Figure 4, C and D). Conversely, although pregnancy rate was improved after Tconv cell transfer (Figure 4A), Tconv cells were ineffective in protecting pregnant dams from fetal loss (Figure 4B) and did not improve fetal weight, placental weight, or fetal/placental ratio compared to dams given RU486 without transferred cells (Figure 4, C and D). Additionally, Tconv cells caused an increase in placental weight, leading to a decreased fetal/placental weight ratio compared with control mice (Figure 4D). Thus CD4^+^CD25^+^ Treg cells, but not Tconv cells, are effective in improving the quality of pregnancy outcome following disruption of luteal P4 signaling. These data demonstrate that Treg cells are causal in mediating adverse pregnancy outcomes after impaired P4 signaling in early pregnancy and that restoring Treg cells is sufficient to alleviate the effects of insufficient P4 signaling.

### Treg cell transfer restores abnormal placental development in mice with impaired luteal phase P4 signaling.

**Figure 5:**
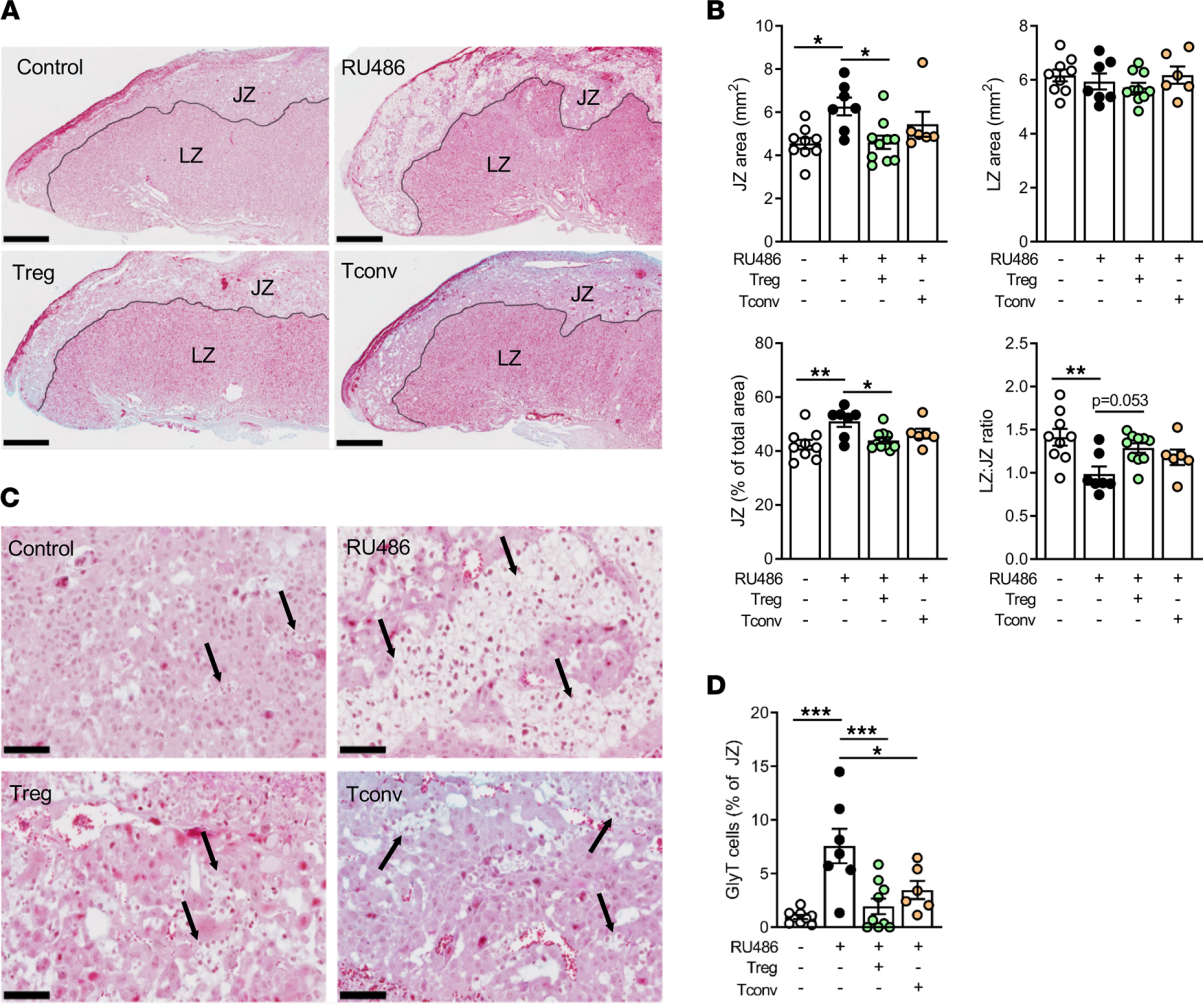
Treg cell transfer restores placental defects caused by impaired luteal phase P4 signaling. Female B6 mice were mated to BALB/c males and administered RU486 (1 mg/kg) or vehicle (control) on 1.5 and 3.5 dpc. On 3.5 dpc, approximately 8 hours following the final RU486 dose, females were injected i.v. with 2 × 10^5^ Treg cells (CD4^+^CD25^+^), Tconv cells (CD4^+^CD25^–^), or vehicle control (PBS). Placentas of fetuses from mice on 18.5 dpc were collected, processed, and stained with Masson’s trichrome to visualize the labyrinth zone (LZ) and junctional zone (JZ). (**A**) Representative midsagittal sections of placentas with labeled LZ and JZ and the LZ-JZ boundary indicated by line. Scale bar = 500 μm. (**B**) The midsagittal cross-sectional area of JZ and LZ (mm^2^), JZ proportion (%total area), and LZ/JZ ratio were quantified. (**C**) Representative midsagittal sections of placentas showing clusters of glycogen trophoblast (GlyT) cells in the JZ, identified by their morphological appearance (indicated by arrows). Scale bar = 100 μm. (**D**) GlyT cell proportion (% of JZ) was quantified. (**B** and **D**) *n* = 6–9 pregnant dams/group with 2 placentas per dam randomly selected for histological analysis. Data shown as mean ± SEM with average values for individual mice indicated by symbols, analyzed by 1-way ANOVA with Sidak’s post hoc *t* test, *P < 0.05, **P < 0.01, ***P < 0.001.

As Treg cells are required for robust placental development (48, 65, 66), we next examined placental structure in dams administered RU486, with or without adoptively transferred Treg cells or Tconv cells. RU486 treatment caused placental structure to be altered on 18.5 dpc, with an increase in junctional zone (JZ) area and a reduced labyrinth zone (LZ) to JZ ratio (Figure 5, A and B). The enlarged JZ was associated with increased abundance of glycogen trophoblast (GlyT) cells (Figure 5, C and D), a specialized type of trophoblast that migrate from the placental JZ to the decidua in late gestation and release stored glycogen to promote rapid fetal growth during this period (67–69). GlyT cells are readily identifiable in placental sections due to their characteristic morphological appearance and vacuolated cytoplasm. Increased retention or delayed migration of GlyT cells in late gestation is a common feature of fetal growth restriction in mice (67, 69) and likely contributes to the fetal growth restriction phenotype caused by reduced luteal P4 signaling. Transfer of Treg cells restored the JZ area and proportion and normalized JZ GlyT cell numbers (Figure 5, A–D). Tconv cells did not restore JZ area but did moderately alleviate GlyT cell accumulation (Figure 5, A–D). These data show that Treg cell dysfunction after reduced P4 signaling modifies normal placental development and structure, notably affecting GlyT cell deployment.

### Treg cell transfer mitigates late gestation loss of udLN Treg cells caused by luteal phase P4 signaling disruption.

**Figure 6:**
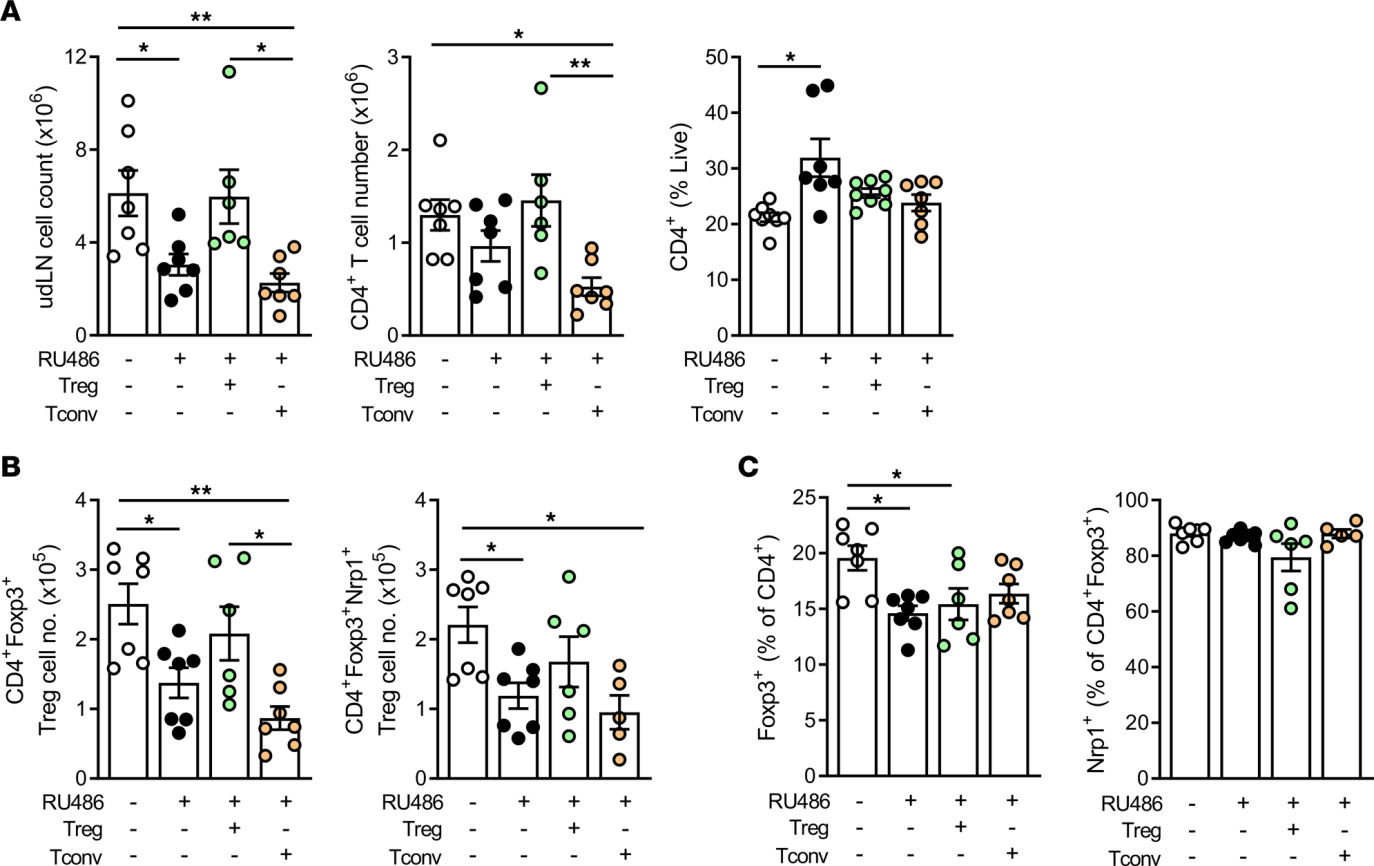
Treg cell transfer mitigates T cell imbalance in late gestation caused by impaired luteal phase P4 signaling. Female B6 mice were mated to BALB/c males and administered RU486 (1 mg/kg) or vehicle (control) on 1.5 and 3.5 dpc. On 3.5 dpc, approximately 8 hours following the final RU486 dose, females were injected i.v. with 2 × 10^5^ Treg cells (CD4^+^CD25^+^), Tconv cells (CD4^+^CD25^–^), or vehicle control (PBS), and then CD4^+^ T cells in udLNs recovered on 18.5 dpc were analyzed by flow cytometry. (**A**) Total cell count, and number and proportion of CD4^+^ T cells, for each group in control mice and mice treated with RU486, RU486+Treg cells, and RU486+Tconv cells. (**B**) Total Treg cell number and Nrp1^+^ Treg cell number. (**C**) Proportion of Foxp3^+^ Treg cells (%CD4^+^) and proportion of Nrp1^+^ (%Treg) per group. (**A**–**C**) *n* = 6–7 pregnant dams/group. Data shown as mean ± SEM with individual mice indicated by symbols, analyzed by 1-way ANOVA with Sidak’s post hoc *t* test, *P < 0.05, **P < 0.01.

T cells are emerging as significant determinants of parturition events and postnatal outcomes. Furthermore, elevated Teff T cells in gestational tissues is a common co-occurrence and potential pathophysiological factor in fetal growth restriction and placental dysfunction. Therefore, we also measured the impact of RU486, with or without adoptively transferred Treg cells or Tconv cells, on the maternal T cell compartment in udLNs on 18.5 dpc. As occurred in midgestation, LN cell numbers were reduced following RU486 treatment (*P* < 0.05) (Figure 6A). Notably, Treg cell transfer restored udLN cell number to control levels, whereas Tconv cell transfer did not, and instead caused a further decline in CD4^+^ T cells (Figure 6A). Treg cells were particularly deficient after RU486 treatment, with a 2-fold decrease in number persisting to late gestation (*P* < 0.05). Both Nrp1^+^ thymus-derived and Nrp1^–^ peripherally induced Treg cells were similarly affected (Figure 6, B and C). Early pregnancy Treg cell transfer restored late gestation Treg cell numbers to control levels but did not alleviate the altered proportion among CD4^+^ T cells (*P* < 0.05), implying elevated Foxp3^–^ Tconv cells are not completely normalized by Treg cell replacement. In contrast, Tconv cell transfer caused Treg cell numbers to decline further compared with control (*P* < 0.01) and Treg cell–transferred (*P* < 0.05) groups. These data suggest that a reduced Treg/Tconv cell ratio in late gestation could contribute to the adverse effects of luteal phase insufficiency on pregnancy survival and fetal growth and indicate that effects of P4 on Treg cells in early pregnancy are important for sustaining maternal immune tolerance into late gestation, with consequences for perinatal outcomes.

### Treg cell transfer mitigates the defect in decidual vessel remodeling in mice with luteal phase P4 signaling disruption.

Recent evidence highlights a key role for Treg cells in supporting the uterine vascular adaptations that must occur in early pregnancy to underpin robust placental development and fetal growth (48, 49). We therefore investigated whether effects on the uterine decidual vasculature contribute to the mechanism by which altered Treg cells mediate the effects of disrupted P4 signaling on placental development, by measuring morphology of decidual blood vessels at 9.5 dpc in implantation sites in a second cohort of RU486-treated dams with transferred Treg or Tconv cells. Generally the pregnancy outcomes were similar to the first Treg cell transfer experiment, with RU486 causing pregnancy loss, and dams given Treg cells exhibiting a pregnancy rate comparable to control mice, in contrast to dams given Tconv cells, which had a 40% lower pregnancy rate (*P* < 0.05) (Supplemental Figure 8A). In pregnant dams, RU486 again reduced the number of viable implantation sites, and Treg cells and Tconv cells both partially attenuated this (Supplemental Figure 8, B and C). Early fetal loss was not accompanied by effects on ovarian P4 synthesis, as plasma P4 at 9.5 dpc was not altered by RU486 treatment or T cell transfers (Supplemental Figure 8D).

**Figure 7:**
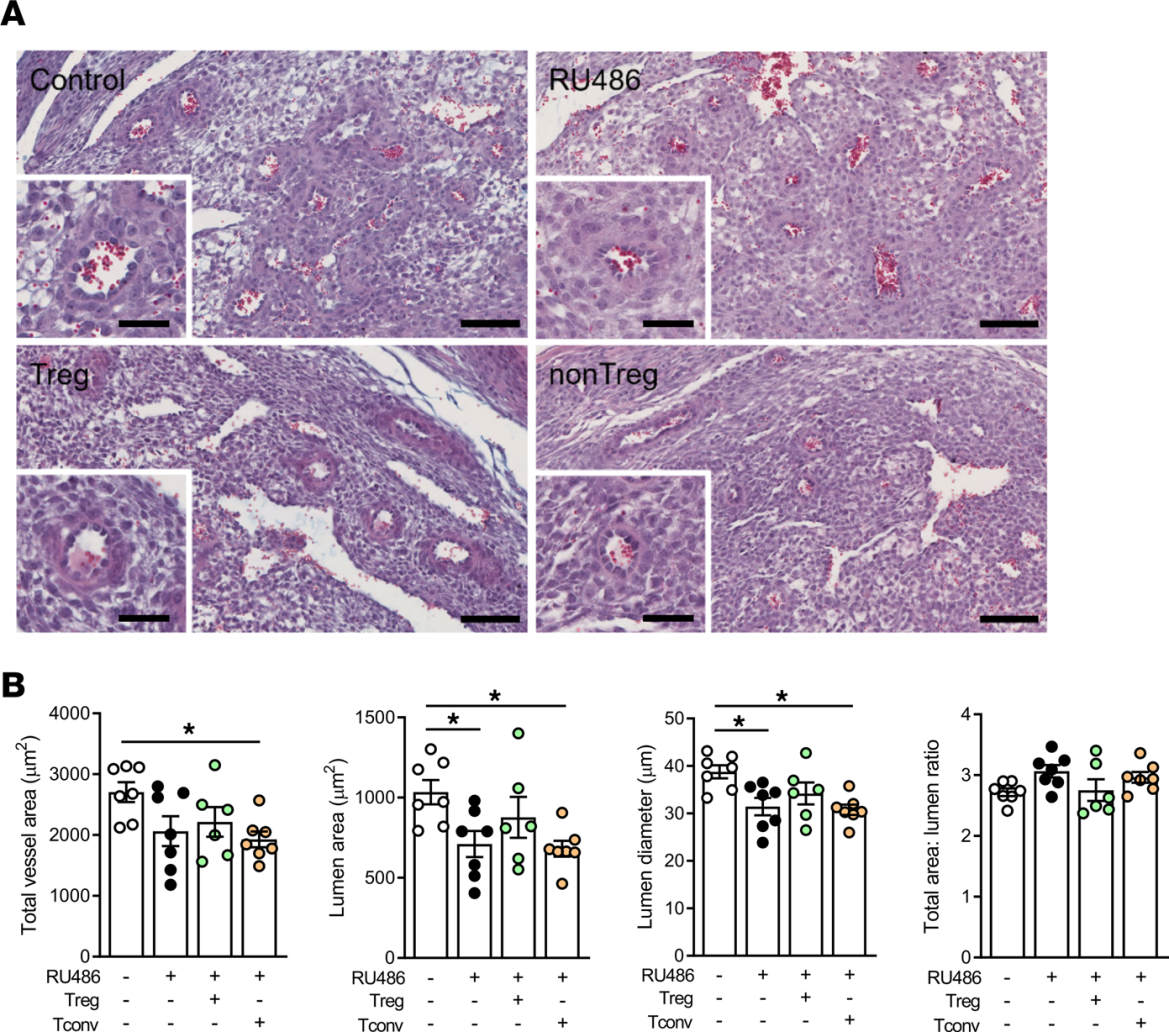
Treg cells mitigate defective decidual vessel remodeling caused by impaired luteal phase P4 signaling. Female B6 mice were mated to BALB/c males and administered RU486 (1 mg/kg) or vehicle (control) on 1.5 and 3.5 dpc. On 3.5 dpc, approximately 8 hours following the final RU486 dose, females were injected i.v. with 2 × 10^5^ Treg cells (CD4^+^CD25^+^), Tconv cells (CD4^+^CD25^–^), or vehicle control (PBS), and then decidual blood vessels were analyzed by histology in implantation sites collected on 9.5 dpc. (**A**) Representative images of cross sections of decidua stained with Masson’s trichrome to enable evaluation of decidual blood vessels. Scale bar = 100 μm (main images); scale bar = 50 μm (insets). (**B**) Total decidual vessel area, diameter, lumen area, and total/lumen area ratio were compared between groups. *n* = 6–7 pregnant dams/group were analyzed, with 1 implantation site per dam randomly selected for analysis. Data are shown as mean ± SEM with individual mice indicated by symbols. Data were analyzed by 1-way ANOVA with Sidak’s post hoc *t* test, *P < 0.05.

RU486-treated mice exhibited perturbation in the structure of decidual blood vessels characterized by reduced vessel diameter and lumen area (*P* < 0.05) compared to control vessels (Figure 7, A and B). Notably, the defect in decidual vessel remodeling was mitigated by Treg cell transfer, resulting in a normal vessel diameter and lumen area that was comparable to controls (Figure 7, A and B). Conversely, transfer of Tconv cells led to a 30% decrease in total vessel area, and the reduced diameter and lumen area were retained (all *P* < 0.05) (Figure 7, A and B). This result shows that Treg cells mediate effects of P4 on decidual vessel remodeling and implies that the ability of Treg cells but not Tconv cells to rescue pregnancy outcomes and fetal growth in RU486-treated mice is associated with their ability to promote uterine vascular adaptation and healthy placental function.

## Discussion

**Figure 8:**
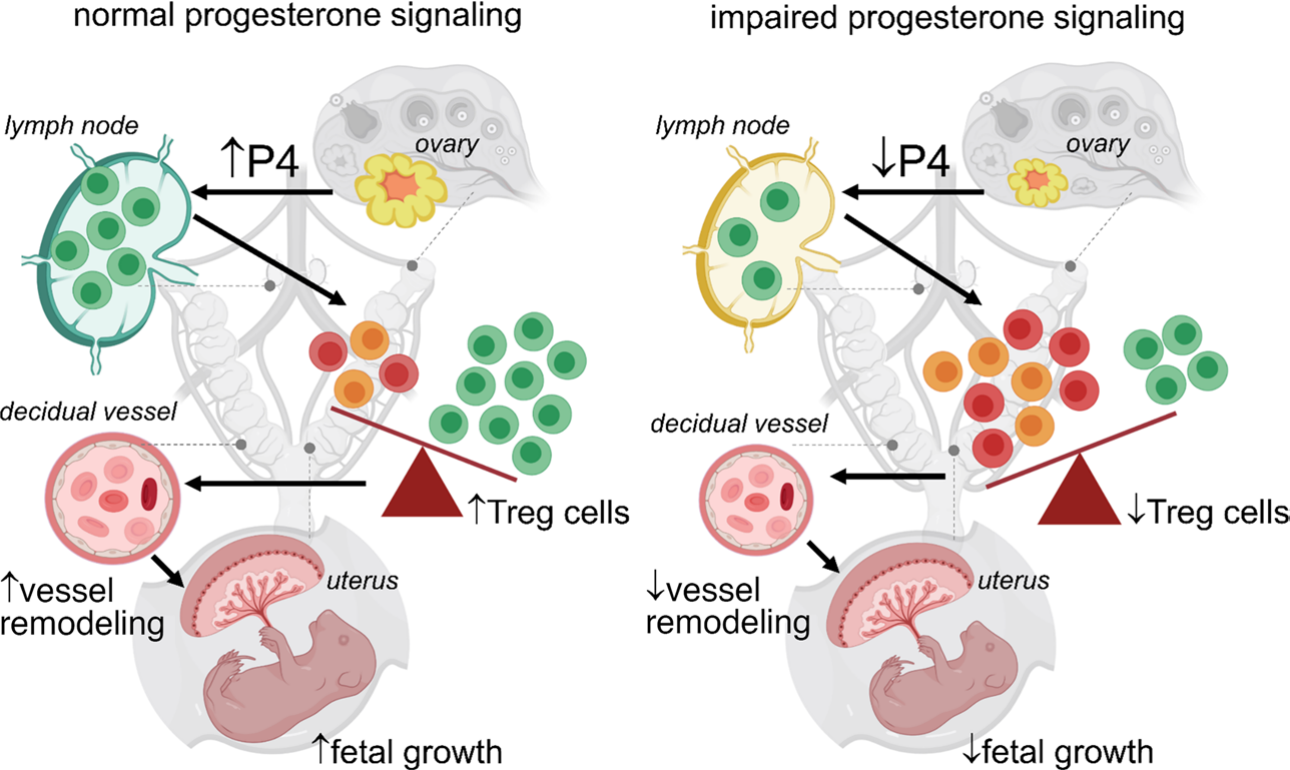
Diagram illustrating effects of impaired P4 signaling on Treg cell generation, the Treg/Tconv cell ratio, decidual vessel remodeling, and fetal growth. In healthy pregnancy, P4 synthesized by the corpus luteum promotes Treg cell proliferation in LNs draining the uterus, to ensure a high Treg cell/Tconv cell ratio in the uterus at implantation and during placental development. This is associated with extensive remodeling of decidual blood vessels to increase placental access to maternal blood and ensure healthy fetal growth. In the case of impaired P4 signaling induced by RU486 administration, Treg cell proliferation is impaired, and the Treg cell/Tconv cell ratio in the uterus is reduced. In turn, this is associated with impaired remodeling of decidual blood vessels and reduced fetal growth. Green lymphocytes = Treg cells; orange and red lymphocytes = Tconv cells. Created with BioRender.com.

There is now strong evidence that events at conception and embryo implantation shape the course of pregnancy progression, with substantial consequences for fetal survival and growth and perinatal outcome (70, 71). As well as factors intrinsic to the embryo, this is due to adaptations in the maternal endocrine and immune response that affect uterine receptivity to embryo implantation and modulate placental development (9, 10, 72). In the current study, we demonstrate that Treg cells, critical mediators of maternal immune tolerance required for implantation and robust placental development, are highly sensitive to P4 bioavailability in early pregnancy. We show that impaired implantation and altered placental development resulting from dysregulated P4 signaling in the luteal phase is primarily attributable to reduced P4-mediated expansion of the CD4^+^Foxp3^+^ Treg cell pool and diminished Treg cell suppressive function. Strikingly, transfer of Treg cells from pregnant donors at implantation was sufficient to mitigate the adverse impact of reduced P4 signaling on midgestation pregnancy loss and to improve fetal weight, showing that of the range of P4-mediated adaptations for pregnancy, Treg cell generation is among the most sensitive to perturbation. Treg cell transfer restored the effect of reduced P4 signaling on uterine vascular adaptation and placental structure, implying the effects of limited Treg cells on fetal survival and growth were largely mediated via compromised placental development secondary to uterine Treg cell deficiency. Therefore, adequate early pregnancy P4 is essential to drive generation of Treg cells necessary for healthy placental development and fetal growth (Figure 8). These findings demonstrate that Treg cells comprise a pivotal effector mechanism through which P4 actions are exerted in early pregnancy to ensure optimal pregnancy success.

The effects of P4 on the mouse uterus at implantation are well characterized (6), and RU486 is a potent inhibitor of many uterine P4-responsive genes that are regulated by genomic PR action (73). These genes include immune mediators (73), consistent with strong evidence that the immune response is a major element of P4-mediated induction of endometrial receptivity, as recent sequencing studies highlight. In women, many of the genes that are differentially expressed from the early to mid-luteal (secretory) phases are immune or inflammatory regulators (74), and single-cell sequencing shows uterine T cells and other immune cells undergo dynamic transcriptional changes in the luteal phase (75). Immune cell and immune-regulatory genes also account for the majority of transcriptional changes as uterine receptivity is acquired in mice (76). However, since T cells comprise a low proportion of the immune cells in the uterus, Treg cell–associated genes can be difficult to discern in global gene expression analyses of P4-regulated changes in the uterine transcriptome.

Treg cells are known to be essential to mediate fetal-maternal immune tolerance and suppress uterine inflammation in early pregnancy (10, 36, 37, 55); however, the biological factors regulating Treg cells are not fully resolved. P4 has been shown to promote Treg proliferation and survival and to limit inflammatory Th1 and Th17 cell responses in mice (50, 51, 53, 63) and is thought to modulate peripheral blood Treg cells in women (77). This raised the question of whether P4 bioavailability during the luteal phase might affect the quality of the Treg cell pool at implantation and later in pregnancy. Here, we show that P4 signaling in the luteal phase is a critical determinant of Treg cell abundance and suppressive competence in mid- and late gestation and that when Treg cells are disrupted, increased CD4- and CD8-associated type 1 immunity arises and causes fetal loss. Our results are consistent with a recent report that targeted mutation of PR in murine Treg cells causes fetal demise by midgestation associated with elevated effector CD8^+^ T cell immunity (18) and supports earlier speculations that Treg cells are causal in RU486-induced pregnancy loss (52). Importantly, our study extends understanding by demonstrating that the effects of P4 on Treg cells are among the most sensitive and crucial of the biological pathways by which P4 acts, and not only are pivotal for establishing pregnancy, but also have consequences for placental development that affect fetal growth and survival much later in gestation.

We found that P4 regulation of both CD4^+^ T cells and Treg cell phenotype is associated with a direct effect of P4 in suppressing IFNG expression. This is consistent with elevated effector CD8^+^ T cell immunity and implantation failure following targeted mutation of PR in Treg cells (18), and several other studies showing that P4 dampens Th1 immunity via the IFNG axis in CD4^+^ and CD8^+^ T cells (53, 63, 78). Since IFNG production in Treg cells is associated with increased plasticity toward Th1-like phenotype and potential loss of Treg stability (79), it seems likely that P4-mediated suppression of IFNG is a key means of reinforcing Treg phenotype commitment.

Several studies indicate that Treg cells influence implantation success and ongoing fetal development (36–38), but the significance of luteal phase P4 in conferring the capacity for Treg cells to exert their positive effects has not been appreciated. Inflammatory activation and elevated CD8^+^ T cells and Th1 cells reacting to fetal antigens are well known to cause fetal loss (40, 80). In healthy pregnancy, this is suppressed when there are sufficient Treg cells to dampen Teff phenotypes and maintain the Foxp3^–^ Tconv population in an anergic state (38, 81). In line with this mode of action, we found transfer of Treg cells restored T cell numbers and repaired the phenotype balance in late pregnancy, implying that P4 dysregulation at implantation has a lasting impact on maternal adaptive immune tolerance for the duration of gestation. Since an altered maternal T cell balance with a shift away from Treg cells and toward Teff cells is implicated in preeclampsia and fetal growth restriction (32–35), this is consistent with the conception environment being instrumental in the pathophysiological origins of these conditions (7, 16) and supports the inference that luteal phase P4 is a contributing factor in their etiology (7, 82).

The shift toward an inflammatory state that occurred due to reduced P4 bioavailability after RU486 administration was associated with increased Th1 and Tc1 cells both locally in udLNs and, to a lesser extent, systemically as reflected in the spleen. The LNs draining the uterus, where Treg cells selectively proliferate in early pregnancy (37, 60, 61) prior to recruitment into the uterus (56), are exposed to very high concentrations of P4 delivered from the ovarian vein into the afferent lymphatics via an unusual countercurrent mechanism (83). The T cells proliferating in response to pregnancy-associated antigens are thus uniquely positioned to sense and respond to perturbation in P4 bioavailability. Although P4 induces transcriptional changes in T cells that suppress Th1 cell induction directly (53, 63, 84), our data support P4 acting to constrain Th1 and Tc1 cell generation primarily by promoting the proliferation and suppressive function of Treg cells. Given that these localized effects of P4 would not be recapitulated by exogenous P4 administration, it seems plausible that the elevated preeclampsia incidence seen in women undergoing assisted reproduction treatment protocols that circumvent corpus luteum development might be explained by adverse impacts on Treg cell generation (16, 85).

The finding that CD4^+^CD25^–^ Tconv cells not only were insufficient to mitigate the effects of reduced P4 signaling, but also led to a further reduction in pregnancy rate and fetal viability, fits with the well-known negative impact of Th1 cells in pregnancy (80). In the absence of adequate P4, Tconv cells presumably adopt a Th1-like phenotype, as was evident in RU486-treated mice at midgestation and in late gestation. This interpretation is supported by the in vitro findings that P4 constrains Th1-type responses. Another explanation is the resistance of Treg cells to cell death caused by physiological levels of P4, compared with Tconv cells, which may be insufficiently curtailed when P4 signaling is limited (53). Only one parameter was improved by Tconv cells — like Treg cells, they normalized the number of GlyT cells in the placenta at 18.5 dpc. It is not clear why this occurred, but it is possible the effect on GlyT cells reflects a mechanism not restricted by T cell phenotype.

Peri-implantation P4 signaling has been reported to influence birth weights in humans (13) and animals (86), but these earlier studies did not define mechanisms or consider Treg cells as potential mediators. Vascular adaptation to pregnancy is essential for correct placental development and fetal growth, and disturbed vascular adaptation causes pathologies in animal models reminiscent of preeclampsia. Treg cells are emerging as important cells capable of influencing the remodeling of the uterine vasculature required for optimal placental development (47), without which placental blood flow and fetal growth are compromised (48, 49). That Treg cells protected against the adverse effects of impaired luteal phase P4 signaling on decidual vessel remodeling supports the interpretation that Treg cells mediate key actions of P4 on placental development and fetal growth via this mechanism.

Impaired decidual vessel adaptation is associated with altered placental structure in other models of fetal growth restriction in mice (69, 87). In a rat model of fetal growth restriction, reduced maternal uteroplacental blood flow and placental hypoxia led to accumulation of GlyT cells in the JZ (88). Constraint of Teff cells is likely to be one mechanism by which Treg cells facilitate vascular remodeling, since Teff cells cause vascular dysfunction in inflammatory vascular conditions such as atherosclerosis (89, 90). This concurs with reports that peripheral Treg cell deficiency impairs spiral artery remodeling and placental inflammation associated with increased decidual Teff cells (66), while in rats Th17 cells induce fetal growth restriction and increase blood pressure (91). Furthermore, Treg cells interact with uNK cells and several other leukocyte lineages in the implantation site that promote uterine vascular adaptations to support placental function (10).

Our data are consistent with P4 modulation of Treg cells via both indirect and direct mechanisms of action. The in vitro findings of P4 modulation of CD4^+^ T cell phenotype through suppressing IFNG expression and Th1 generation are in line with other evidence of T cell–intrinsic effects of P4 (63, 64, 92). Recent work suggests the main receptor mediating actions of P4 in CD4^+^ T cells is PR (18, 64), though glucocorticoid receptor (20, 53, 93) and membrane progesterone receptor (94) could also be involved. This phenotype-skewing effect may not require PR expression in T cells — indirect, PR-dependent regulation of uterine Treg cell abundance and phenotype during pregnancy may occur via P4 effects on antigen-presenting cells (20) and/or nonimmune uterine cell lineages (95, 96). An important mechanism of P4 modulation of Treg numbers during pregnancy is via P4 effects in the thymus. Thymic involution orchestrated by PR-expressing thymic stromal cells decreases thymic T cell output from early pregnancy (97, 98), potentially limiting or skewing output to favor Treg cells (28, 99, 100). tTreg cells form the majority of the udLN Treg compartment in early pregnancy (60), and our finding that they are particularly sensitive to disrupted P4 signaling could reflect effects in the thymus. Lymphatic endothelial cells also express various P4 receptors and control P4 bioavailability in vitro by metabolizing the hormone (101). Together with the high concentration of P4 found in the afferent lymphatics of the udLNs in early pregnancy (83), thymic and vascular effects may be other means by which the expanding Treg cell pool is exposed to P4 effects during phenotype commitment in early pregnancy.

The finding that Treg cells are highly sensitive to limited P4 bioavailability contrasts with the relative tolerance of the uterine endometrium to low P4 concentrations. That implantation occurred normally when Treg cells were replaced indicates that other P4-dependent aspects of receptivity were not affected by low-dose RU486 treatment. This is consistent with 2 studies in women (102, 103) revealing that unexpectedly, histological features of endometrial development are unaffected when levels of P4 are well below those normally observed in the luteal phase of the cycle, and similarly, endometrial gene expression is only altered when levels of P4 are substantially less than the physiological threshold for healthy implantation (102, 103). This lack of sensitivity in the endometrial compartment has been a factor in failure to develop a consensus understanding of the mechanisms and diagnostic features of luteal phase deficiency in women (104).

The evolutionary significance of Treg cell sensitivity to P4 is not clear. Since optimal corpus luteum development and P4 synthesis depend on the integrated effect of several factors, including immune and endocrine regulators (2, 105), Treg cell responsiveness to P4 could provide a sensitive mechanism by which environmental conditions can differentially modulate female reproductive investment (106). When favorable conditions promote luteal sufficiency, stronger maternal immune tolerance would maximize the likelihood of optimal placentation, but luteal insufficiency in the event of adverse conditions would impair generation of tolerance to suppress pregnancy progression. Further studies will be required to evaluate this speculation.

It is notable that fetal growth restriction induced by RU486 administration was improved by Treg cell transfer, but fetal weight remained less than control values. This partial effect might be due to technical limitations of the Treg cell transfer approach and the challenge of acquiring and administering sufficient cells to fully correct the Treg cell deficiency. Alternatively, it could reflect the actions of Treg cell–independent, P4-responsive mechanisms by which fetal growth restriction arises after luteal P4 insufficiency. One such mechanism is delayed embryo implantation, which occurs due to slower embryo development and transport following direct and indirect effects of P4 insufficiency on the embryo and the oviduct secretome (107). Direct effects of P4 on the decidual response likely also contribute (108).

Our findings have direct relevance in women with luteal phase deficiency (104) and other forms of infertility. Although immune response genes are typically identified as responsive to P4 levels and associated with acquisition of uterine receptivity in mice and in women, and immune-modulating effects of P4 at implantation are well appreciated (3, 109, 110), luteal phase deficiency has not previously been viewed as having an immune mechanism. Furthermore, exogenous P4 is routinely given to women undergoing in vitro fertilization as luteal phase support, but the degree to which it faithfully recapitulates endogenous P4 effects on immune adaptation is rarely considered (111, 112). Whether Treg cells are directly or indirectly impacted by P4 resistance, caused by altered endometrial stromal cell responsiveness to P4 signaling (113), also requires further evaluation. A better understanding of the significance of the immune response as potentially the most sensitive aspect of P4 regulation of endometrial receptivity will help inform improvements in P4 supplementation for treating infertility and recurrent miscarriage (114), as well as later onset disorders of pregnancy that originate in disorders of maternal immune tolerance.

In summary, the results reported herein provide understanding of the pathophysiological mechanism of luteal phase deficiency and point to a mechanism operating via disrupted Treg cells. The findings provide an appreciation of the significance of luteal phase P4 as a factor in generating the Treg cell defects that contribute to pregnancy complications and infertility in women (25–30) and indicate that investigation of the impact of luteal phase deficiency on Treg cells and fetal growth in clinical cohorts of at-risk women is warranted. A better understanding of the relationship between P4, Treg cells, and placental development will provide biological insight necessary to advance treatments for infertility and obstetric disorders arising from failure of maternal immune adaptation at the outset of pregnancy.

## Methods

### Animals.

C57BL/6J female and male mice, B6.SJL-Ptprca Pepcb/BoyJ (CD45.1) female mice, and BALB/c male mice were housed in specific pathogen–free conditions. Female mice (8–14 weeks old) were housed with proven fertile BALB/c stud males, and the presence of a copulatory plug was designated 0.5 days post coitum (dpc). See Supplemental Methods for details.

### RU486 model of reduced P4 signaling.

RU486 (mifepristone, 17 beta-hydroxy-11 beta-[4-dimethylaminophenyl]-17 alpha-[1-propynyl]estra-4,9-dien-3-one; MilliporeSigma) was administered to mated B6 females on 1.5 and 3.5 dpc at 0.5, 1, 2, 4, or 8 mg/kg. Control mice were administered vehicle. Pregnant mice were defined by the presence of ≥1 implantation site (at 9.5 dpc) or fetuses (at 18.5 dpc). Pregnancy rate (%) was calculated as (number of pregnant mice/number of mated mice) × 100. See Supplemental Methods for details.

### Flow cytometry.

Single-cell suspensions from spleen and LNs were preincubated to elicit cytokine expression, stained to detect surface markers, and permeabilized and stained for detection of intracellular markers, according to standard protocols using fluorophore-conjugated antibodies (Supplemental Table 1). Data were acquired using FACSDiva Software and analyzed using FlowJo software with a standardized gating strategy (Supplemental Figure 9). See Supplemental Methods for details.

### T cell isolation and adoptive transfer.

CD4^+^CD25^+^ (Treg) or CD4^+^CD25^–^ (Tconv) cells isolated from the spleen and LNs of BALB/c-mated B6 or CD45.1 females on 11.5–14.5 dpc were adoptively transferred by i.v. injection into RU486-treated B6 mice about 8 hours following the final RU486 injection on 3.5 dpc. See Supplemental Methods for details.

### In vitro T cell differentiation.

Splenocytes from female B6 mice in estrus were cultured with α–mouse-CD3 and α–mouse-CD28 under Th0-, Th1-, or Th17-polarizing conditions (Supplemental Table 2) in the presence or absence of P4 (4-pregnene-3, 20-dione, 0.5 μg/mL), then restimulated for 4 hours before FACS staining and analysis. See Supplemental Methods for details.

### Placental and decidual histology.

Formalin-fixed, paraffin-embedded 9.5 dpc implantation sites and 18.5 dpc placentas were sectioned and stained with Masson’s trichrome using standard protocols and analyzed using NDP.view2 software. Decidual vessels were analyzed in 9.5 dpc implantation sites by NDP.view2 software. See Supplemental Methods for details.

### Treg cell suppression assay.

Isolated CD4^+^CD25^+^ Treg cells were incubated with CFSE-labeled responder Tconv (CD4^+^CD25^–^) cells from spleens, in the presence of CD3/CD28 activation. CFSE content in responder cells was analyzed by flow cytometry at 96 hours. See Supplemental Methods for details.

### Progesterone assay.

Serum P4 concentration was measured using the ALPCO Mouse/Rat Progesterone ELISA kit according to manufacturer’s instructions. See Supplemental Methods for details.

### Statistics.

Statistical analysis was performed using 1-way ANOVA with Sidak’s post hoc *t* test and χ^2^ test (GraphPad Prism 8). Linear mixed model ANOVA in SPSS Statistics 25 was used to evaluate fetal and placental weight data. See Supplemental Methods for details.

### Study approval.

All animal experiments were approved by the University of Adelaide Animal Ethics Committee (approval 31874) and conducted in accordance with the Australian Code of Practice for the Care and Use of Animals for Scientific Purposes (8th edition, 2013).

## Author contributions

ESG designed and conducted experiments, acquired and analyzed data, interpreted data, and wrote the manuscript. LMM contributed to the study design and interpreted data. HMG and DJS conducted experiments and acquired and analyzed data. PYC conducted experiments and analyzed data. ASC designed experiments and interpreted data. RLR and SRM contributed to research study design, provided reagents, and interpreted data. SAR devised and oversaw the study, interpreted data, wrote the manuscript, and secured funding. All authors reviewed and edited the manuscript.

## Supplementary Material

Supplemental data

## Version 1 05/16/2023

In-Press Preview

## Version 2 06/08/2023

Electronic publication

## Version 3 06/13/2023

The values of RU486 administered to mice were updated in the abstract
