# Supplementary material for: Regulatory T cells are paramount effectors in progesterone regulation of embryo implantation and fetal growth
Source: JCI Insight. 2023 Jun 8;8(11):e162995. doi: 10.1172/jci.insight.162995 (PMC10393240; doi:10.1172/jci.insight.162995)
Supplement: Supplemental data [file jciinsight-8-162995-s282.pdf]

Supplementary Information for

Regulatory T cells are paramount effectors in progesterone regulation of embryo implantation and fetal growth

Authors: Ella S. Green[1], Lachlan M. Moldenhauer[1], Holly M. Groome[1], David J. Sharkey[1], Peck Y. Chin[1], Alison S. Care[1], Rebecca L. Robker[1], Shaun R. McColl[2] and Sarah A. Robertson[1‡]

[1]Robinson Research Institute and Adelaide Medical School, The University of Adelaide, Adelaide, SA 5005, Australia.

[2]School of Biological Sciences, University of Adelaide, Adelaide, SA 5005, Australia.

Corresponding author:
Sarah A Robertson PhD, Robinson Research Institute, Adelaide School of Medicine, University of Adelaide, Adelaide SA 5005, Australia. T: +61 8 8313 4094, E-mail: sarah.robertson@adelaide.edu.au

This PDF file includes:

Supplementary Material and Methods
Supplementary Figures 1 to 10
Supplementary Tables 1 to 2

SI Materials and Methods

Animals

C57Bl/6J female and male mice, B6.SJL-Ptprca Pepcb/BoyJ (CD45.1) female mice, and BALB/c male mice were purchased from Animal Resource Centre (ARC, Perth, Australia). All mice were housed in specific pathogen-free conditions at the University of Adelaide Medical School Animal House (MSAH) or CSIRO barrier research facilities. All mice were kept on a 12 h light/dark cycle and food and water were provided ad libitum. Mice were fed a diet of 10% fat chow (Envigo, Cambridgeshire, UK).
For all mating experiments, female mice (8-14 weeks old) were housed with proven fertile BALB/c stud males overnight. When a copulatory plug was detected the following morning this was designated day 0.5 post-coitum (pc). The estrous cycle was tracked by daily analysis of vaginal smears and females in proestrus were selected for mating (1). For in vitro experiments requiring non-pregnant mice, females in estrus were used.

RU486 model of reduced P4 signaling

RU486 (mifepristone, 17 beta-hydroxy-11 beta-[4-dimethylaminophenyl]-17 alpha-[1-propynyl] estra-4,9-dien-3-one; Sigma-Aldrich, St Louis MO, USA) was administered subcutaneously to mated B6 females on 1.5 and 3.5 dpc at various doses (0.5, 1, 2, 4 or 8 mg/kg). Stock RU486 (20 mg/mL in ethanol) was diluted in sesame oil (Sigma-Aldrich) to concentrations (0.1-1.6 mg/mL) to provide the specified mg/kg doses in 5 µL/g body weight. Control mice were administered vehicle alone (sesame oil containing 1% ethanol). For most experiments, mice were administered 1 mg/kg RU486 or vehicle and maternal/fetal outcomes were assessed on 9.5 dpc, 18.5 dpc, or postpartum. Pregnant mice were defined by the presence of ≥1 implantation sites (at 9.5 dpc) or fetuses (at 18.5 dpc). Pregnancy rate (%) was calculated as [number of pregnant mice/ number of mated mice] x 100. Outcomes on 9.5 dpc were recorded as the number of total, viable, or abnormal implantation sites; on 17.5 dpc as viable, non-viable, or resorbing fetuses; and at birth as the number of viable pups. At 9.5 dpc, abnormal implantation sites were defined as small (<2 mm) and/or avascular. At 18.5 dpc, resorbing fetuses were defined as small (<4 mm) hemorrhagic masses, and non-viable fetuses were severely growth restricted and/or avascular, compared to viable fetuses (2). Fetal viability (%) was calculated as [number of viable implantations (or fetuses)/ total number of implantations (or fetuses)] x 100. On 18.5 dpc viable fetuses and placentae from each litter were dissected from the uterus and weighed.

Flow cytometry

Spleen, uterus draining para-aortic lymph nodes (udLN) and other lymph nodes (inguinal, brachial, axillary, mesenteric) were excised and prepared into single cell suspensions. Spleens were homogenized by gentle crushing through a 70 µm cell strainer (BD Biosciences) using the plunger end of a 5 mL syringe (BD Biosciences, Biosciences, San Jose CA, USA) into a petri dish containing complete RPMI-1640 media (cRPMI; RPMI + 10% FBS and 2% penicillin/streptomycin (Gibco, Grand Island NY, USA)). Cells were washed in media by centrifugation then erythrocytes were lysed by incubating in 5 mL of pre-warmed RBC lysis buffer (0.155 M NH_4Cl, 10 mM $KHCO_3$, 99.2 µM EDTA disodium salt in RO water) at 37°C in for 5 min followed by addition of 5 mL cRPMI. LNs were gently crushed between the frosted ends of two SuperFrost glass slides (HD Scientific Supplies Pty Ltd, Australia) into a petri dish containing cRPMI. Suspensions were transferred to 15 mL tubes, washed and resuspended for cell counting. 1-1.5 x 10^6 cells were added to 96-well U bottom plates (Corning Inc., Corning NY, USA; Cat#9377) for cytokine stimulation. Cells were washed and resuspended in 50 µL cRPMI supplemented with 20 ng/mL phorbol 12-myristate 13-acetate (PMA; Sigma), 1 nM ionomycin (Life Technologies, Waltham MA, USA) and 1/1500 dilution of Golgi Stop (as per manufacturers instruction, BD Biosciences). Cells were incubated for 4 h at 37°C in 5% CO_2. Following stimulation, cells were washed and stained with fixable viability dye 620 (BD Biosciences; 1/1000 dilution) for 20 min at RT in the dark. Cells were washed 2x in FACS buffer (PBS + 0.1% BSA/0.05%

sodium azide) and resuspended in 50 μL α-Fc-cIIR antibody (Fc block; BD Biosciences; 1/100 dilution) in FACS buffer at RT for 15 min in the dark. A master-mix of fluorophore-conjugated antibodies against surface markers (Supplemental Table 1) diluted in Brilliant Stain Buffer per well (BD Biosciences) was added to cells to reach the final antibody concentrations specified. Samples were incubated for 25 min in the dark at 4°C, washed 2x then fixed and permeabilized using the Foxp3 Staining Buffer Set (eBiosciences, Thermo Fisher Scientific, Waltham MA, USA). For intracellular staining, cells were washed in 1 x permeabilization wash and stained with fluorophore-conjugated antibodies against intracellular markers (Supplemental Table 1) diluted in permeabilization wash. Samples were incubated in the dark at 4˚C for 30 min, washed 2x in FACS buffer and resuspended in PBS for analysis, or resuspended in PBS containing 1% paraformaldehyde for analysis up to 5 days post-staining. Data was acquired on a BD Canto II, BD Aria III or BD LSR Fortessa X-20, using FACS Diva Software. Single stain, unstimulated and fluorescence minus one (FMO) controls were included in each experiment. Data was analyzed using FlowJo software (Treestar, Ashland, OR, USA) with a standardized gating strategy (Supplemental Figure 10).

T cell isolation and adoptive transfer
$CD4^+CD25^+$ (Treg cells) or $CD4^+CD25^-$ (Tconv) T cells were isolated from spleen and lymph nodes (LNs) of BALB/c mated donor B6 or CD45.1 females on 11.5-14.5 dpc using EasySep™ mouse regulatory T cell isolation kit, according to the manufacturer's instructions (Stemcell Technologies Cat# 18783, Vancouver, Canada). The purity of isolated cell suspensions was determined by flow cytometry staining (between 80-95%). Treg and Tconv cell suspensions were adoptively transferred to RU486-treated B6 mice ~8 h following the final RU486 injection on day 3.5 pc. Treg or non-Treg cells were suspended in sterile PBS at 2 x 10^6 cells/mL and 2 x 10^5 cells (100 μL) were administered intravenously via tail vein injection. In experiments using CD45.1 donor females, the presence of donor Treg or Tconv cells was confirmed in the udLN of recipients 72 h post-transfer by flow cytometry.

In vitro T cell differentiation
2x 10^5 splenocytes isolated from female B6 mice in estrus were cultured in complete Iscove's Modified Dulbecco Media (cIMDM; IMDM + 10% FBS, 100 U/mL penicillin/streptomycin and 0.002 M L-glutamine (Gibco)) under Th0-, Th1- or Th17- polarizing conditions as previously described (3), in the presence of absence of P4 (4-pregnene-3, 20-dione, 0.5 μg/mL). α-mouse-CD3 (clone 2C11) Ab (10 μg/mL in PBS, 50 μL per well) was added to 96-well U-bottom cell culture plates (Corning Inc., Cat#9377) and incubated at 37°C for 90 min followed by 2x washes in 200 μL 1 x PBS. T helper (Th) cell polarizing media for non-polarizing (Th0-) conditions, and Th1- and Th17-polarising conditions were prepared in cIMDM with the addition of α-mouse CD28 (2 μg/mL). Cytokines and neutralizing antibodies used are described in Supplemental Table 2. Th-polarizing media containing P4 or vehicle (100 μL) was added to wells, followed by addition of 2 x 10^5 splenocytes/well in 100 μL cIMDM. Cells were cultured for 48 h at 37°C in 5% CO_2. Cells were then washed 2x in PBS and restimulated for 4 h before FACS staining and analysis (as described above).

Placental and decidual histology
Implantation sites on 9.5 dpc and placentas on 18.5 dpc were fixed in 10% neutral-buffered formalin (Australian Biostain, Australia) for 24 h followed by two 1 x PBS washes for 24 h, and stored in 70% ethanol until processing. Tissues were dehydrated in a graded ethanol series and embedded in paraffin using a Leica TP1020 Tissue Processor (Leica Microsystems, Wetzlar, Germany). Tissues stored at -20°C were sectioned to 6 μm on a Leica Rotary Microtome (Leica Microsystems) and mounted on slides before rehydration and staining. Midsagittal cross-sections of placental tissue (2 placentas/dam) and cross-sections of implantation sites were stained with Masson trichrome using standard protocols, imaged on a Nanozoomer-XR Digital Slide Scanner (Hamamatsu Photonics, Hamamatsu, Japan) and analyzed using NDP.view2 Viewing software (Hamamatsu Photonics). Cross-sectional areas of placental junctional zone (JZ) and labyrinth zone (LZ) were measured on digital images of complete

midsagittal sections of placentas on 18.5 dpc. The proportion (%) of each region in the placenta was estimated by dividing the cross-sectional area (mm^2) of that region by the total midsagittal cross-sectional area of the placenta. Glycogen trophoblast cells (GlyT) of these placentae were identified and quantified as % JZ region based on their distinctive appearance (4).

Several viable and abnormal implantation sites were assessed at 9.5 dpc for the presence of a viable embryo or signs of fetal loss, characterized by absence of a fetus and degenerating or necrotic fetal tissue. Decidual vessels were analyzed morphometrically on 9.5 dpc in one viable implantation site (exhibiting normal fetal development)/dam. Vessels within the middle two quadrants of the decidua, closest to the central plane in reference to embryo location, were analyzed as described (5). Freehand region tracing and ruler functions in NDP.view2 software were used to measure total vessel circumference and area (including the lumen and wall) and vessel lumen circumference and area. The total vessel area: lumen area ratio and vessel diameter (of the internal lumen) were then calculated.

Treg cell Suppression Assay

Mated female mice received RU486 (1 mg/kg) or vehicle control on 1.5 and 3.5 dpc, before the udLN were harvested from pregnant mice on 8.5-9.5 dpc, and prepared into a single cell suspension. $CD4^+CD25^+$ Treg cells were isolated from udLN using EasySep^{TM} mouse regulatory T cell isolation kit, as above. $CD4^+CD25^+$ cells were pooled from mice if required to obtain the cell number necessary. $CD4^+CD25^+$ cells were placed into wells of a 96 well cell culture plate (Corning Inc.; Cat#3799) and serially diluted 2 in 1, from 20,000 cells down to 312.5 cells per well, plus control wells containing no $CD4^+CD25^+$ cells, in a total final volume of 40 μl / well of suppression assay media [RMPI 1640 (Gibco) + 1/100 dilution of anti-anti antibiotic-antimycotic (Gibco) + 50 μM β-mercaptoethanol (Sigma-Aldrich) + 10% FBS (Gibco). Spleens were harvested from unmated B6 female mice, a single cell suspension generated and RBCs lysed, as described above. Responder Tconv ($CD4^+CD25^-$) cells were isolated from spleens using the EasySep^{TM} mouse regulatory T cell isolation kit, as per the manufacturer's instructions. Responder Tconv cells were counted and labelled with carboxyfluorescein succinimidyl ester using the CellTrace^{TM} CFSE Kit (ThermoFisher Scientific; Cat#C34554), as per the manufacturer's instructions. Responder Tconv cells were seeded at 10,000 cells / well in suppression assay media containing Mouse T-Activator CD3/CD28 Dynabeads^{TM} (Gibco), following manufacturer's instructions, to activate responder $CD4^+CD25^-$ Tconv cells, with the final volume made up to 50 μl / well. Viable $CD4^+CFSE^+$ responder cells were assessed for proliferation 96 h later by analysing CFSE proliferation peaks by flow cytometry and the extent of proliferation determined by calculating the Proliferation Index using FlowJo software for each sample. In order to normalise the extent of proliferation and suppression in each mouse, every experiment had control wells which contained activated responder cells and lacked Treg cells (responder:Treg ratio of 1:0). These control wells were given the value of 100% proliferation, to which all other responder:Treg ratios from that mouse were compared to determine the extent of suppression. The mean and standard deviation at each ratio was plotted and t-test was used to compare groups at each ratio.

Progesterone Assay

Serum was recovered from blood collected from terminally anaesthetized control, RU486-, Treg- and Tconv-treated mice and stored at -80°C. Serum P4 concentration was measured using ALPCO Mouse/Rat Progesterone ELISA kit (ALPCO, Salem NH, USA) according to manufacturers' instructions. Assay sensitivity was 0.4 ng/mL. This kit has a detection range of 0.4-100 ng/mL and reports minimal cross-reactivity with other steroid hormones. Serum was diluted in kit diluent and samples were run in duplicate.

Statistics

Statistical analysis was performed using GraphPad Prism 8 for Windows (GraphPad software, San Diego CA, USA). For data comparing two groups, unpaired T-tests were performed. Data comparing

four groups was analyzed by one-way ANOVA with Sidak post-hoc t-test. ROUT's test was used to identify outliers greater than two standard deviations from the mean, which were excluded from analysis. To analyze categorical data (e.g. pregnant vs non-pregnant) Chi-squared test was performed. Fetal/placental weight data on 18.5 dpc was analyzed using SPSS Statistics 25 (SPSS Inc., Chicago IL, USA) using Linear Mixed Model ANOVA, with dam as the subject, and data are presented as violin plots to depict median and quartile values from individual dams. All other data are presented as mean ± SEM (standard error of mean) with scatter plots using symbols to depict data from individual mice. Statistical significance was set at $P \leq 0.05$.

Study Approval

All animal experiments were approved by the University of Adelaide Animal Ethics Committee (approval #31874) and conducted in accordance with the Australian Code of Practice for the Care and Use of Animals for Scientific Purposes 8th edition 2013.

References

1. Byers SL, Wiles MV, Dunn SL, and Taft RA. Mouse estrous cycle identification tool and images. *PLoS One.* 2012;7(4):e35538.
2. Pang SC, Janzen-Pang J, Tse MY, Croy BA, and Lima PDA. In: Croy BA, Yamada AT, DeMayo FJ, and Adamson SL eds. *The Guide to Investigation of Mouse Pregnancy*. Boston: Academic Press; 2014:3-19.
3. Kara EE, Comerford I, Bastow CR, Fenix KA, Litchfield W, Handel TM, et al. Distinct chemokine receptor axes regulate Th9 cell trafficking to allergic and autoimmune inflammatory sites. *The Journal of Immunology.* 2013;191(3):1110-7.
4. Akison LK, Nitert MD, Clifton VL, Moritz KM, and Simmons DG. Review: Alterations in placental glycogen deposition in complicated pregnancies: Current preclinical and clinical evidence. *Placenta.* 2017;54:52-8.
5. Kieckbusch J, Gaynor LM, Moffett A, and Colucci F. MHC-dependent inhibition of uterine NK cells impedes fetal growth and decidual vascular remodelling. *Nat Commun.* 2014;5:3359.

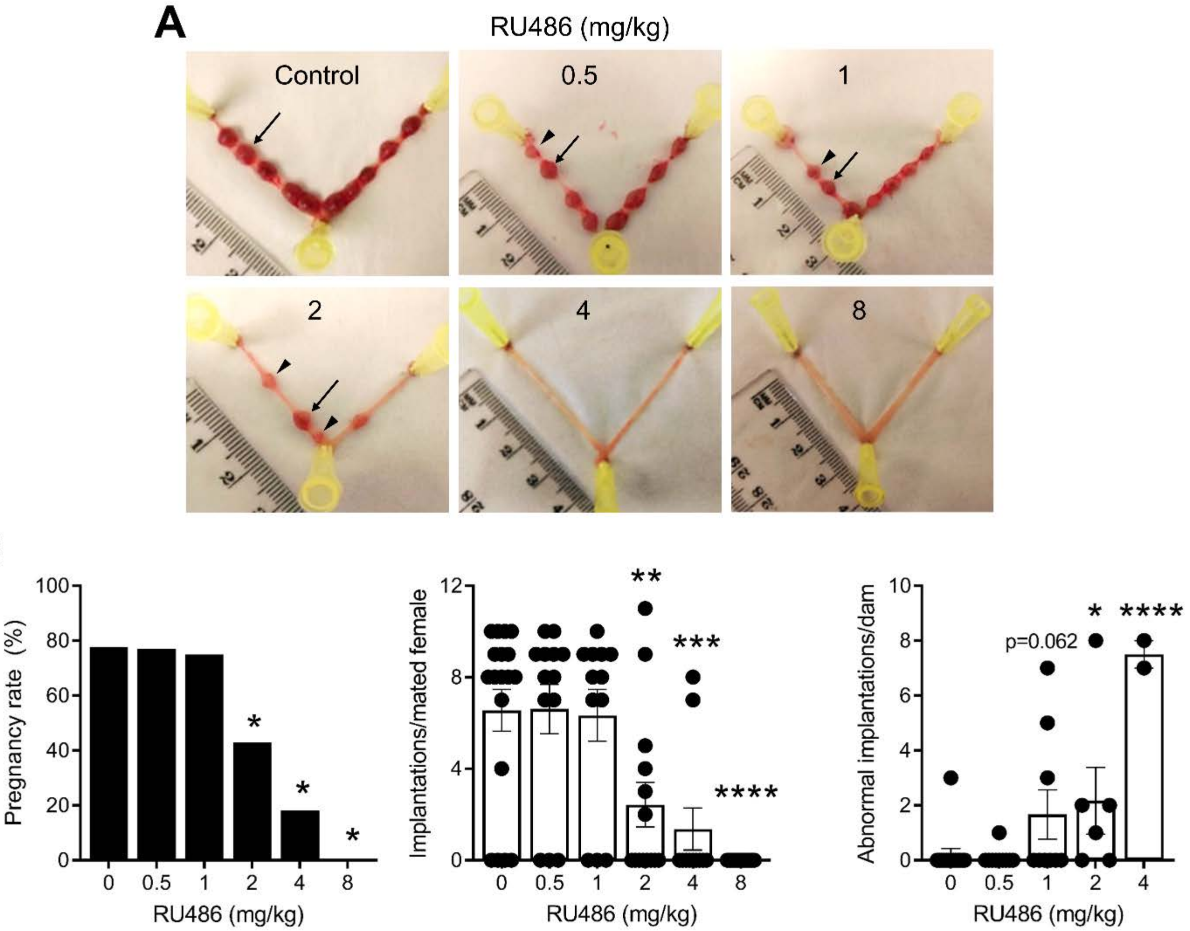

Supplemental Figure 1. Impaired luteal phase progesterone signaling elicits a dose-dependent effect on implantation. Female C57Bl/6 (B6) mice were mated to BALB/c males and administered RU486 (0.5 - 8 mg/kg) or vehicle (control) on 1.5 and 3.5 dpc, and pregnancy was assessed on 9.5 dpc. **(A)** Appearance of uteri from RU486-treated mice. Large arrows indicate viable implantation sites, small arrowheads indicate abnormal implantation sites, identified as small (<2 mm) and/or avascular. **(B)** Pregnancy rate (%mated mice with ≥1 viable implantation site), total number of implantation sites per mated female, and number of abnormal implantation sites per pregnant dam. Data from Figure 1 is duplicated here for 0 and 1 mg/kg groups. Pregnancy rate was analysed by Chi-squared test. *n* = 14-18 mated females per group. Number of total and abnormal implantations is shown as mean ± SEM, with individual mice indicated by symbols, and was analyzed by unpaired *t*-test. *$P < 0.05$, **$P < 0.01$, ***$P < 0.001$, ****$P < 0.0001$, #$P < 0.1$.

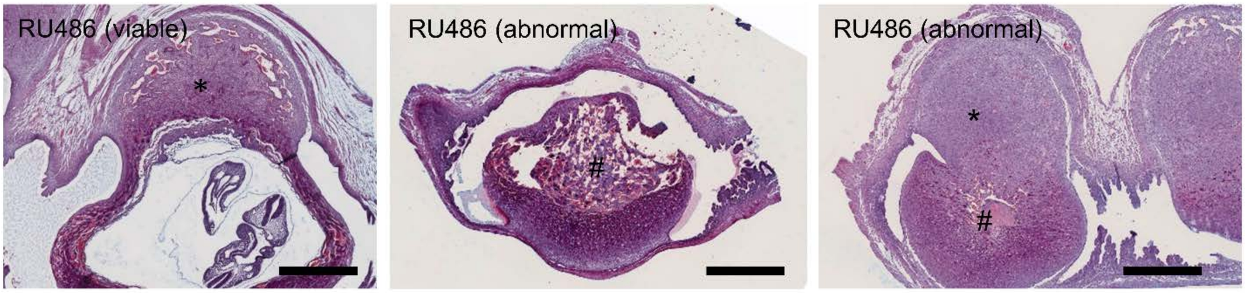

Supplemental Figure 2. Histological analysis of fetal demise at 9.5 dpc following impaired luteal phase P4 signaling. Female C57Bl/6 (B6) mice were mated to BALB/c males and administered RU486 (1 mg/kg) or vehicle (control) on 1.5 and 3.5 dpc, and pregnancy was assessed on 9.5 dpc. Representative photomicrographs of implantation sites from RU486-treated mice classified as ‘viable’ showing typical decidual mass and normal fetal development, and implantation sites classified as ‘abnormal’ (<2 mm and/or avascular) showing fetal degeneration/necrotic tissue, with typical or atypical decidual tissue. Sections were stained with Masson’s trichrome. Scale bar = 1 mm. *indicates mesometrial decidual tissue; #indicates degenerating fetal tissue. **A r**epresentative photomicrograph of an implantation site from a control mouse is provided in Figure 1.

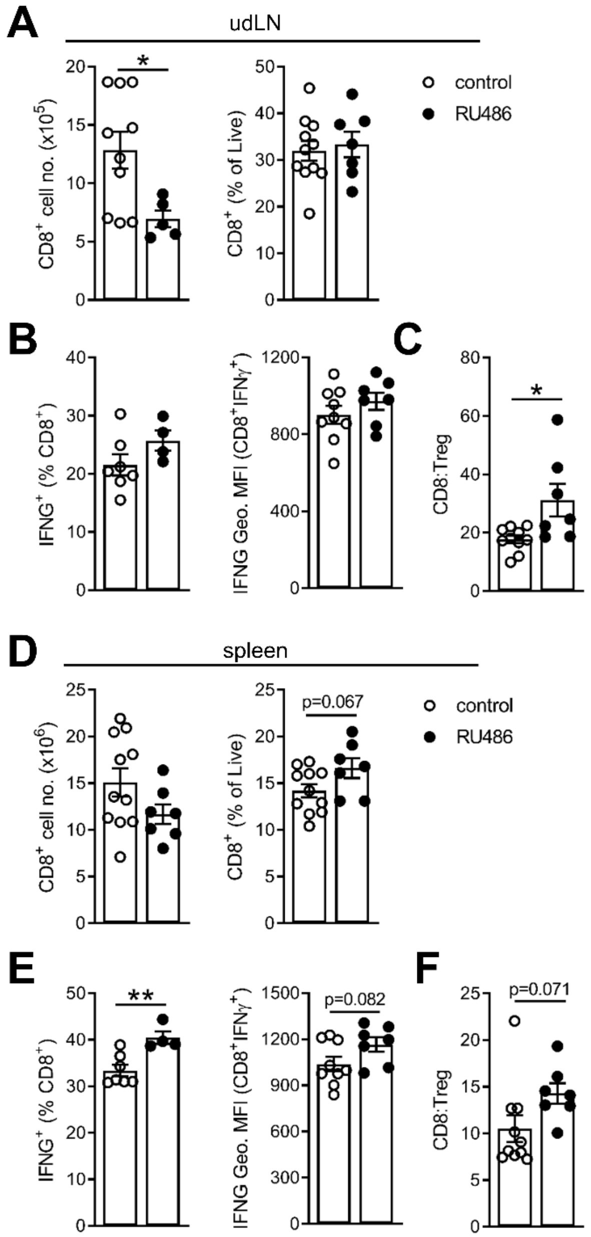

Supplemental Figure 3. Impaired luteal phase P4 signaling perturbs number and phenotype of CD8$^+$ T cells in udLN and spleen in mid-gestation. Female C57Bl/6 (B6) mice were mated to BALB/c males and administered RU486 (1 mg/kg) or vehicle (control) on 1.5 and 3.5 dpc, then udLN were excised from pregnant (≥1 viable implantation site) mice on 9.5 dpc for flow cytometric analysis of CD4$^+$ T cells in the udLN and spleen of vehicle (control) and RU486-treated mice that were pregnant (≥1 implantation site). Cells were stimulated with PMA/Ionomycin for 4 h at 37ºC prior to staining. (**A, D**) Number and proportion of CD8$^+$ T cells in the (**A**) udLN and (**D**) spleen of control and RU486-treated mice. (**B, E**) Proportion of CD8$^+$ T cells expressing IFNG and geometric mean fluorescence intensity (geo. MFI) of IFNG in CD8$^+$IFNG$^+$ cells in (**B**) udLN and (**E**) spleen. The ratio of CD8:Treg cells in (**C**) udLN and (**F**) spleen. *n* = 4-11 pregnant dams/group. Data are shown as mean ± SEM with individual mice indicated by symbols. Data were analyzed by unpaired *t* test; * p<0.05, ** p<0.01.

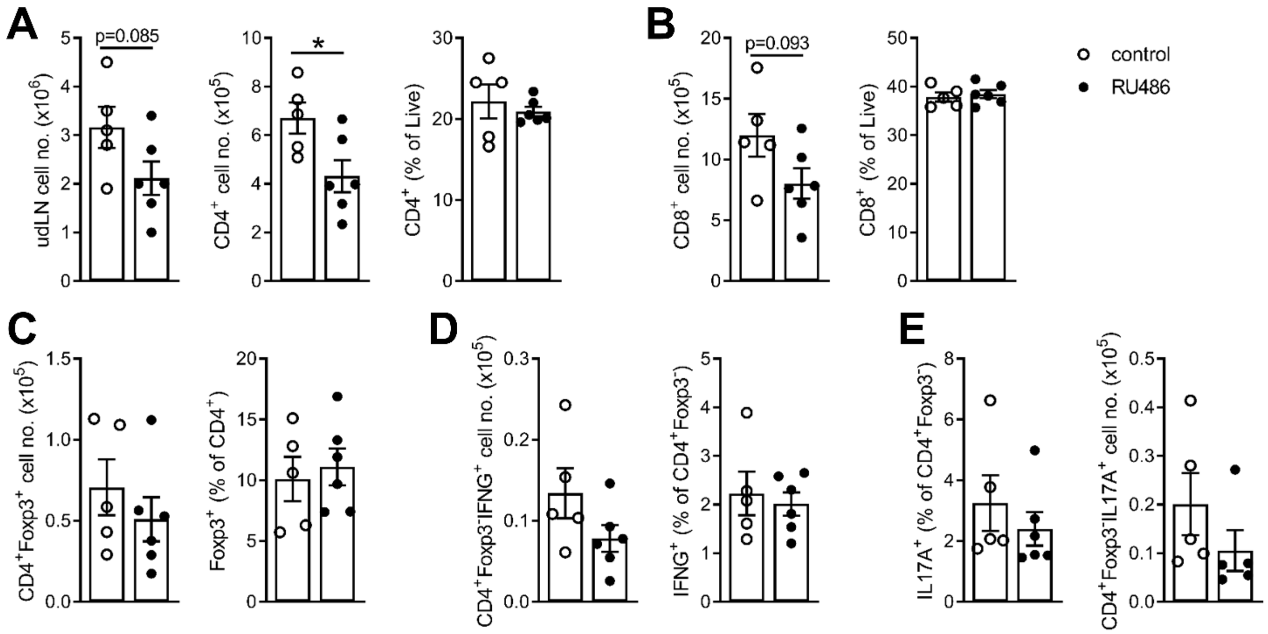

Supplemental Figure 4. Impaired luteal phase P4 signaling reduces udLN CD4⁺ T cell number at implantation. Flow cytometric analysis of CD4⁺ T cells in the udLN of vehicle (control) and RU486-treated mice 24 h following treatment, on day 4.5 post-coitus. Cells were stimulated with PMA/ionomycin for 4 h at 37ºC prior to staining. (**A**) total cell count, and number and proportion of CD4⁺ T cells and (**B**) CD8⁺ T cells in udLNs of control and treated (RU486) mice. (**C**) Foxp3⁺ Treg cell number and proportion (of CD4⁺ cells), (**D**) IFNG⁺ Th1 cell number and proportion (of CD4⁺Foxp3⁻ cells), and (**E**) IL17⁺ Th17 cell number and proportion (of CD4⁺Foxp3⁻ cells) in udLNs of control and RU486 mice. n = 5-6 pregnant dams/group. Data are shown as mean ± SEM with individual mice indicated by symbols. Data were analyzed by unpaired *t*-test; *$P < 0.05$.

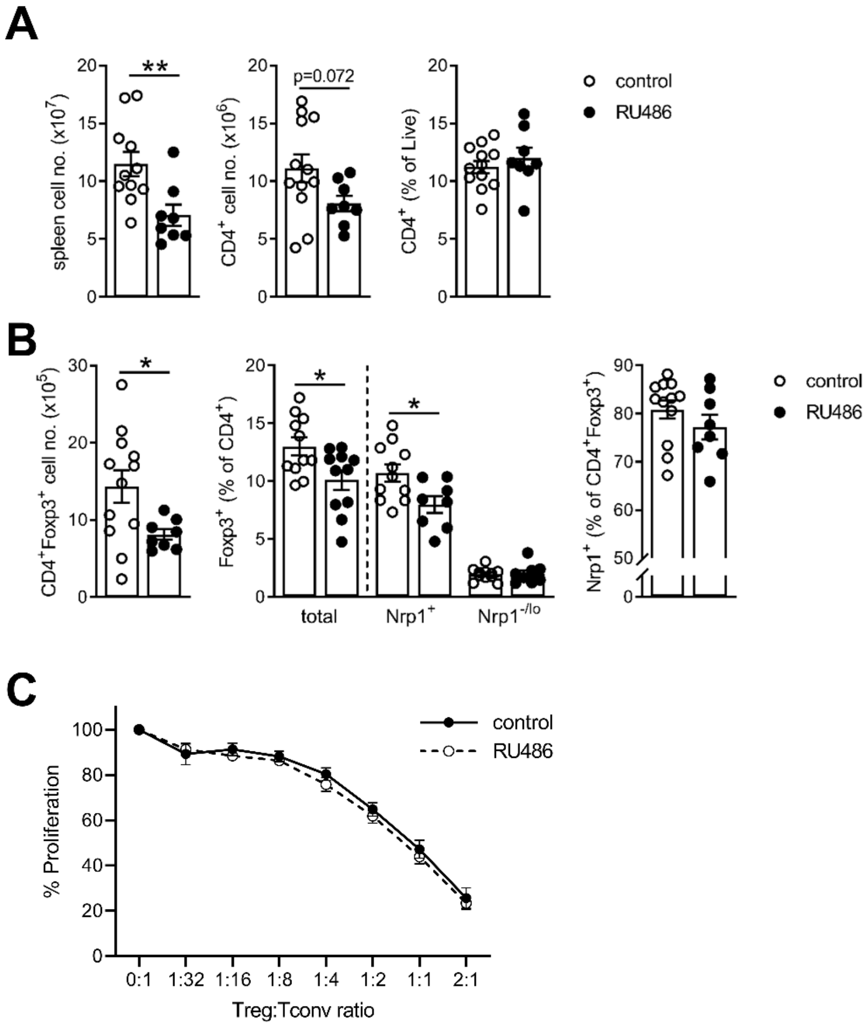

Supplemental Figure 5. RU486-induced impaired luteal phase progesterone signaling causes a reduction in thymic-derived Treg cells in the spleen at mid-gestation. Flow cytometric analysis of CD4$^+$ T cells in the spleen of vehicle (control) and RU486-treated mice that were pregnant (≥1 implantation site) on 9.5 dpc. Cells were stimulated with PMA/ionomycin for 4 h at 37ºC prior to staining. (**A**) total cell count, and number and proportion of CD4$^+$ T cells in the spleen of control and treated mice. (**B**) Foxp3$^+$ Treg cell number, proportion of Foxp3$^+$, Foxp3$^+$Nrp1$^+$ (tTreg) and Foxp3$^+$Nrp1$^{-/lo}$ (pTreg) Treg cells (of CD4$^+$ cells), and proportion of Foxp3$^+$Nrp1$^+$ cells (%Foxp3$^+$ cells). (**C**) Ex vivo analysis of suppressive activity in Treg (CD4$^+$CD25$^+$) cells isolated from spleen of individual pregnant control or RU486-treated mice on 8.5-9.5 dpc, and co-incubated with responder spleen Tconv (CD4$^+$CD25$^-$) cells. Tconv proliferation was determined by CSFE staining and flow cytometry analysis. Proliferation of Tconv cells (%control, no Treg cells) at each Treg:Tconv ratio is depicted. (**A-C**) n = 8-11 pregnant dams/group. Data are shown as mean ± SEM with individual mice indicated by symbols. Data were analyzed by unpaired t-test; *P < 0.05, **P < 0.01.

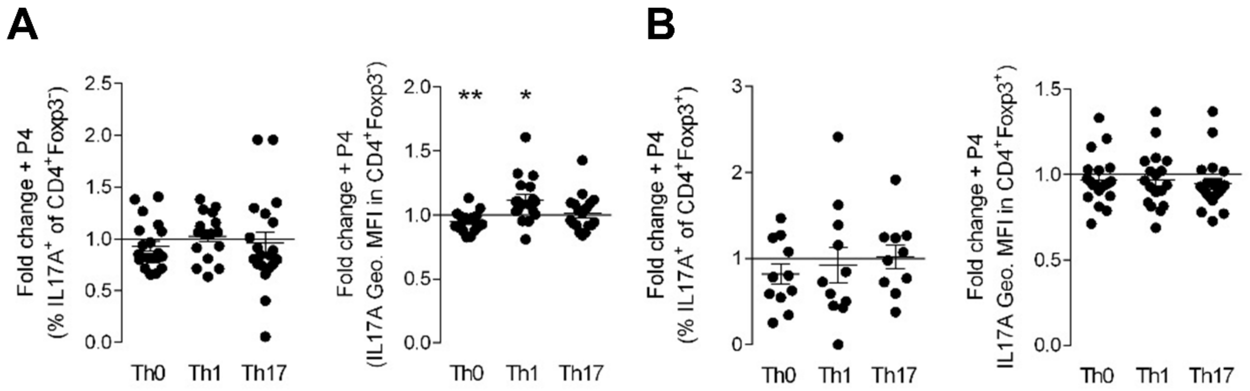

Supplemental Figure 6. P4 does not suppress IL17 production in Teff and Treg cells in vitro. Splenocytes from B6 female mice in estrus were cultured under Th1-polarising, Th17-polarising or non-polarising conditions in the presence (+P4) or absence (-P4) of progesterone (P4) (0.5 µg) for 48 h followed by stimulation with PMA and ionomycin for 4 h and subsequent quantification of T effector (Teff) and Treg cell cytokine production by flow cytometry. Proportion and geometric MFI of IL17A in (**A**) Teff cells and (**B**) Treg cells, expressed as fold change +P4 compared to respective -P4 culture. n = 15-21 pregnant dams/group. Each symbol represents an individual mouse/culture condition. Data are pooled from 5 individual experiments and are shown as mean fold change ± SEM. Data were analyzed by One Sample t-test where -P4 culture = 1; *$P < 0.05$, **$P < 0.01$, ***$P < 0.001$.

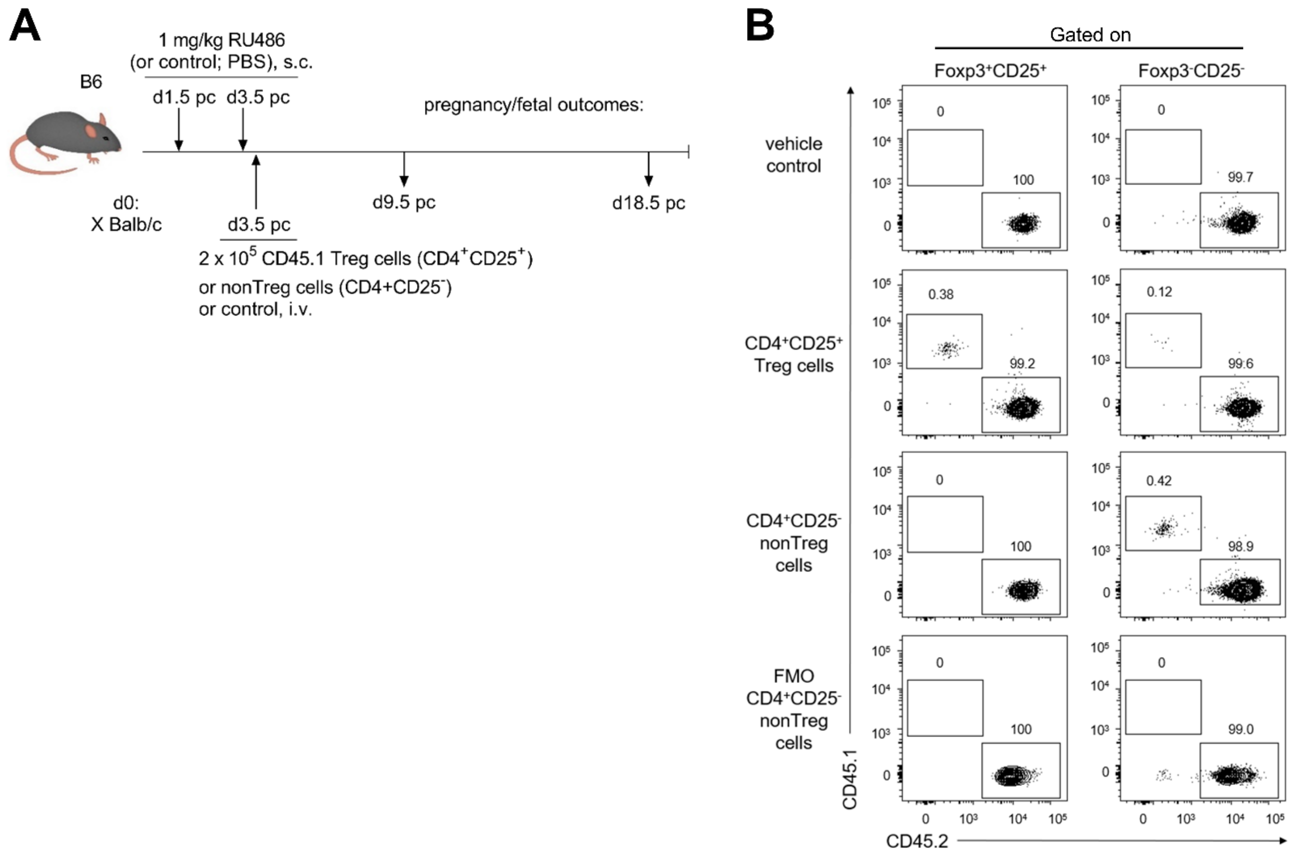

Supplemental Figure 7. Experimental design for adoptive T cell transfer to dams with impaired luteal phase P4 signaling and detection of donor Treg and nonTreg cells in recipient dams. Female B6 mice were mated to BALB/c males and administered RU486 (1 mg/kg) or vehicle (control) on 1.5 and 3.5 dpc. On 3.5 dpc, approximately 8 h following the final RU486 dose, females were injected i.v. with 2 x 10^5 Treg cells (CD4$^+$CD25$^+$), Tconv cells (CD4$^+$CD25$^-$) or vehicle control (PBS). Treg and Tconv cells were enriched from LNs and spleen excised from pregnant donor females on 11.5-14.5 dpc. (**A**) Schematic of experimental design. Pregnancy and fetal outcomes were measured on 9.5 and 18.5 dpc. (**B**) On 6.5 dpc (72 h post transfer) recipient mice were euthanised and donor cells were measured by flow cytometry. Representative FACS plots show udLN Foxp3$^+$ Treg and Foxp3$^-$ Tconv cell populations, in mice transferred with control, Treg cells (CD4$^+$CD25$^+$), and Tconv cells (CD4$^+$CD25$^-$). Donor (CD45.1$^+$) and recipient (CD45.2$^+$) cells among Foxp3$^+$ Treg and Foxp3$^-$ Tconv cell populations were identified using antibodies against CD45.1 and CD45.2. A fluorescence minus one (FMO) control for a mouse transferred with Tconv cells confirms positive staining of donor cells only when the CD45.1 Ab is used.

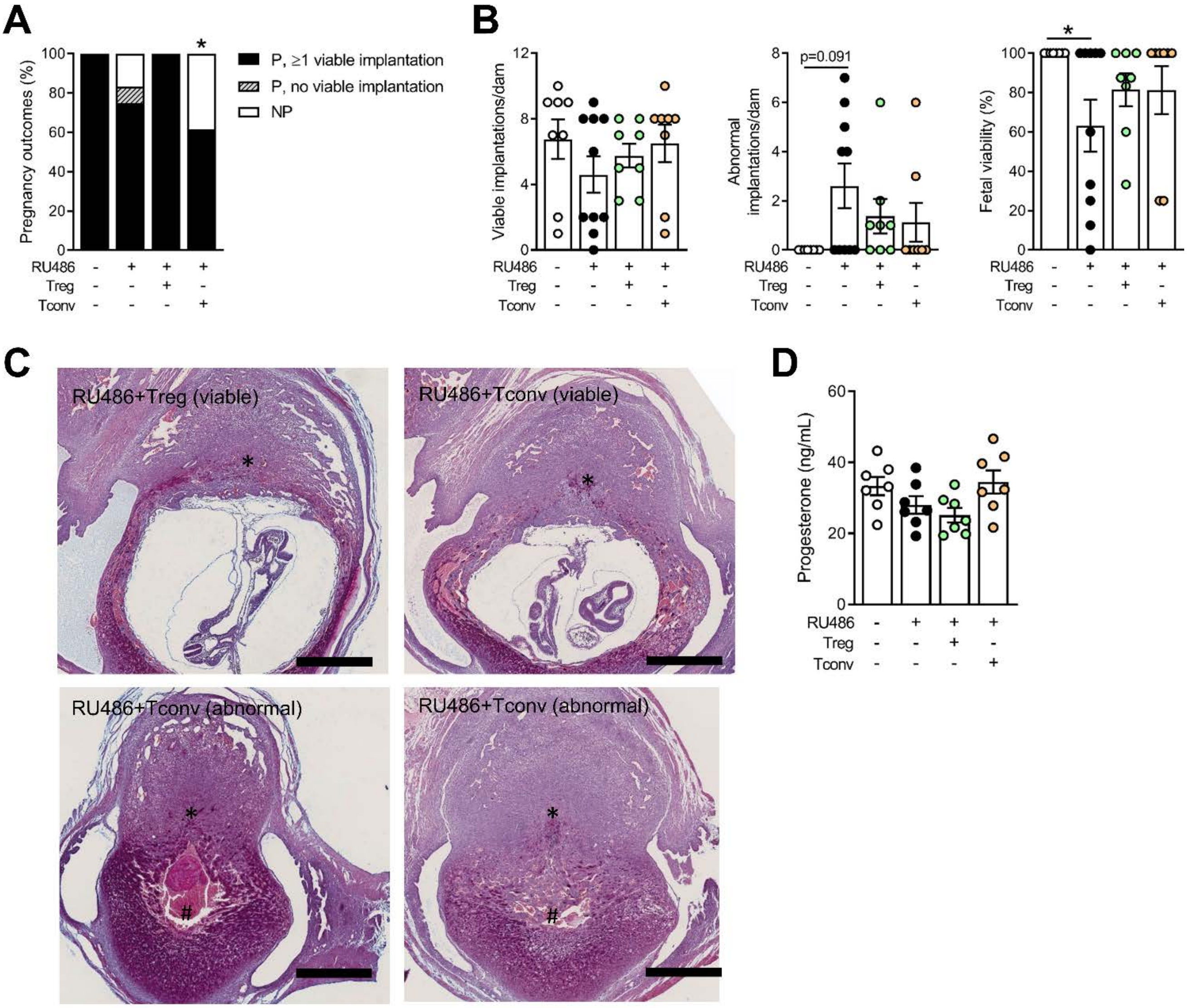

Supplemental Figure 8. Pregnancy progression and plasma P4 concentration at mid-gestation following T cell transfer to dams with impaired luteal phase P4 signaling. Female B6 mice were mated to BALB/c males and administered RU486 (1 mg/kg) or vehicle (control) on 1.5 and 3.5 dpc. On 3.5 dpc, approximately 8 h following the final RU486 dose, females were injected i.v. with 2 x 10^5 Treg cells ($CD4^+CD25^+$), Tconv cells ($CD4^+CD25^-$) or vehicle control (PBS). On 18.5 dpc maternal and fetal outcomes were measured. (**A**) Pregnancy outcomes for dams treated with control, RU486, RU486+Treg cells, or RU486+Tconv cells, classified as pregnant with ≥1 viable fetus, pregnant with only non-viable fetuses, or non-pregnant. P = pregnant; NP = non-pregnant. (**B**) Number of normal (viable) implantation sites per pregnant dam, number of abnormal implantation sites (fetal losses) per pregnant dam, and fetal viability as %total implantation sites per pregnant dam, were measured. (**C**) Representative photomicrographs of implantation sites at 9.5 dpc from dams treated with Treg cells or Tconv cells. Sections are stained with Masson's trichrome for histological examination of fetal viability. Scale bar = 1 mm. *indicates mesometrial decidua. #indicates degenerating fetal tissue. Representative photomicrographs from control and RU486-treated dams are provided in Figure 1 and Supplemental Figure 2. (**D**) Plasma P4 levels (ng/ml) on 9.5 dpc. (**A, B, D**) Treatment is indicated by -/+ symbols. (**A**) *n* = 8-13 mated mice/group; data analysed by Chi-squared test. (**B, D**) *n* = 7-10 pregnant dams/group. Data is mean ± SEM with individual mice indicated by symbols. Data was analyzed by one-way ANOVA with Sidak post-hoc *t*-test, *$P < 0.05$.

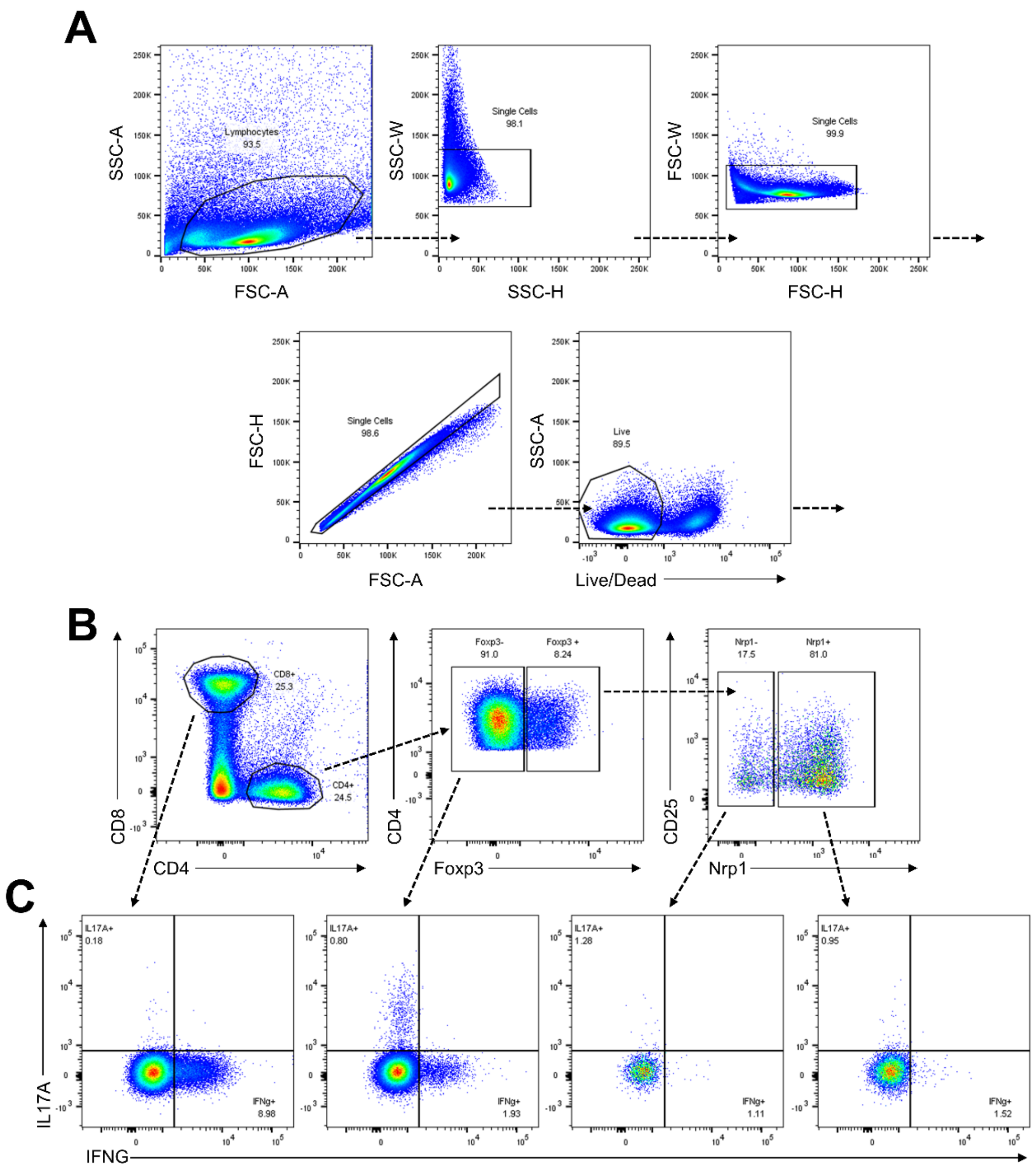

Supplemental Figure 9. Flow cytometry gating strategy used to characterize T cell populations. 1 x 10^6 spleen or LN cells were incubated with live/dead discrimination dye and stained with surface and intracellular antibodies to enable classification of T cell subsets by flow cytometry analysis. (**A**) Lymphocytes were gated based on their forward scatter (FSC-A) and side scatter (SSC-A) characteristics. Doublets were eliminated by SSC and FSC width (-W) vs height (-H) gating followed by FSC-H vs area (-A) proportional gating. Single cells that stained positive for fixable viability dye (Live/Dead) were excluded from analysis. (**B**) T cell subsets were identified based on expression of CD4 and CD8 amongst live cells. Treg cells were defined as $CD4^+Foxp3^+$ cells, and Tconv cells were defined as $CD4^+Foxp3^-$. Treg cell subsets were further classified based on expression of Nrp1. Thymic Treg cells (tTregs) were defined as $Nrp1^+$ and peripheral Treg cells (pTregs) were defined as $Nrp1^{-/lo}$. (**C**) In ex vivo cytokine stimulation experiments, intracellular inflammatory cytokine expression (IFNG and IL17A) was assessed within CD8, Tconv, pTreg and tTreg populations, using quadrant gates.

Supplemental Table 1. Monoclonal antibodies used in flow cytometry experiments.

anti-	Conjugate	Clone	Source	Stock conc. (mg/ml)	Used conc. (µg/mL)
			Surface		
CD4	APC-Cy7	GK 1.5	BD Biosciences (BD)	0.2	2.22
	V450	RM4-5	BD	0.2	0.83
CD45.1	PECy7	A20	eBiosiences (eBIo)	0.2	1
CD45.2	FITC	104	eBio	0.5	2.08
CD25	PE	7D4	BD	0.2	2.22
	FITC	7D4	BD	0.2	2.22
	BB515	PC61	BD	0.2	2.22
CD304 (Nrp1)	V421	3E12	Biolegend	0.025	0.33
CD8	PE-Cy7	53-6.7	BD	0.2	2.22
			Intracellular		
Foxp3	APC	FJK-16s	eBio	0.2	2.22
	PE	R16-715	BD	0.2	3.33
IFNγ	BV510	XMG1.2	Biolegend	0.1	2
	PE-Cy7	XMG1.2	eBio	0.2	1.11
IL17	PE	eBio17B7	eBio	0.2	1.33
	AF647	TC11-18H10	BD	0.2	1

Supplemental Table 2. Cytokines and neutralizing antibodies used in T cell differentiation assays.

Polarizing condition	Reagent type	Reagent (cytokine, antibody, hormone)	Source	Final Concentration	Final dilution
Th0	Neutralizing antibody	IFNγ (XMG1.2)	BD	20 ng/mL	1/357
		IL-4 (11B11)	BD	20 µg/mL	1/411
Th1	Neutralizing antibody	IL-4 (11B11)	BD	20 µg/mL	1/411
	Cytokine	1L-12	R&D systems	20 ng/mL	1/500
Th17	Neutralizing antibody	IFNγ (XMG1.2)	BD	20 ng/mL	1/357
		IL4 (11B11)	BD	20 µg/mL	1/411
	Cytokine	rh TGFb	R&D systems	40 ng/mL	1/500
		IL-6	R&D systems	40 ng/mL	1/250
		rm IL-1β	R&D systems	20 ng/mL	1/500
		rm IL-23	R&D systems	20 ng/mL	1/500
